# Fundamental Limits of Coded Caching in Request-Robust D2D Communication Networks †

**DOI:** 10.3390/e26030250

**Published:** 2024-03-12

**Authors:** Wuqu Wang, Zhe Tao, Nan Liu, Wei Kang

**Affiliations:** 1 National Mobile Communications Research Laboratory, Southeast University, Nanjing 211189, China; wuquwang@seu.edu.cn; 2 Huawei Technologies, Nanjing 210012, China; 3 School of Information Science and Engineering, Southeast University, Nanjing 211189, China

**Keywords:** coded caching, device-to-device, request-robust, order-optimal scheme

## Abstract

D2D coded caching, originally introduced by Ji, Caire, and Molisch, significantly improves communication efficiency by applying the multi-cast technology proposed by Maddah-Ali and Niesen to the D2D network. Most prior works on D2D coded caching are based on the assumption that all users will request content at the beginning of the delivery phase. However, in practice, this is often not the case. Motivated by this consideration, this paper formulates a new problem called *request-robust D2D coded caching*. The considered problem includes *K* users and a content server with access to *N* files. Only *r* users, known as requesters, request a file each at the beginning of the delivery phase. The objective is to minimize the average and worst-case delivery rate, i.e., the average and worst-case number of broadcast bits from all users among all possible demands. For this novel D2D coded caching problem, we propose a scheme based on uncoded cache placement and exploiting common demands and one-shot delivery. We also propose information-theoretic converse results under the assumption of uncoded cache placement. Furthermore, we adapt the scheme proposed by Yapar et al. for uncoded cache placement and one-shot delivery to the request-robust D2D coded caching problem and prove that the performance of the adapted scheme is order optimal within a factor of two under uncoded cache placement and within a factor of four in general. Finally, through numerical evaluations, we show that the proposed scheme outperforms known D2D coded caching schemes applied to the request-robust scenario for most cache size ranges.

## 1. Introduction

In recent years, the demand for user throughput has greatly increased by applications based on fifth-generation (5G) mobile networks [1], such as short videos, self-driving vehicles, the Metaverse, etc. Fortunately, the data of such applications can be pre-stored in the user’s storage during low-network consumption periods, preventing network congestion during peak hours. This approach is known as *caching* [2]. There are typically two phases in the caching process [3]. Before knowing any user requests, the server fills the users’ caches in the *placement phase* during off-peak hours. The *delivery phase* follows in peak hours. The delivery signals will be designed and transmitted from terminals like the server or the users to satisfy all user demands when they are revealed. Technology for caching has advanced quickly in recent years, and it is currently regarded as one of the effective methods for relieving the congestion of wireless networks.

Traditional caching ignores the processing capability of the users, and therefore, the contents cached by the users and the signals transmitted by the server are both uncoded. In contrast to traditional caching, *coded caching* [3], proposed by Maddah-Ali and Niesen, uses a combination of coded multi-casting and device caching to simultaneously fulfill multiple requests through coded transmissions. The coded caching strategy works for the prototypical network topology called the *single shared-link network*, e.g., vehicular networks [4]. Both the cache contents of the users and the delivery signal from the server are allowed to be coded in the coded caching problem. The aim is to design a caching and delivery scheme that minimizes the *average* and *worst-case* delivery rate, which is defined as the average and worst-case number of broadcast bits among all possible user requests. When the optimal caching and delivery scheme that achieves the lowest worst-case delivery rate can be found for any user cache size, the optimal tradeoff between rate and memory for the system is determined. If each user directly stores a subset of the files’ bits in its cache without coding, it is referred to as an *uncoded* cache placement scheme; otherwise, it is referred to as a *coded* cache placement scheme. The original problem [3] studied in coded caching is *centralized*, assuming that all users present during the placement phase will each make a request for a file at the beginning of the delivery phase. *Decentralized coded caching* [5,6,7] also considers the possibility of users leaving or turning off during the delivery phase and explores less coordinated caching strategies.

Taking self-driving vehicles as an example: one promising approach to improve the communication efficiency is through the use of device-to-device (D2D) communication, which allows the users to directly exchange information with each other without the need for a server like a base station. This can be particularly useful in situations where the traditional infrastructure is limited or unavailable, such as in remote or rural areas. To solve the coded caching problem in these scenarios, a framework is proposed by Ji et al. in [8] for D2D coded caching. In the placement phase, similar to coded caching [3], the server fills the users’ caches before the users make any requests. In the delivery phase, when the users reveal their demands, the server is disconnected from the users and it is up to the users to communicate with each other so that each user can decode the file it requested using the signals transmitted by the other users and the contents of its local cache. For the centralized D2D coded caching problem, the caching strategy of [3] (Algorithm 1), which is uncoded, is widely used in the placement phase, e.g., in [8,9] and so on. In [8], a novel delivery scheme was provided that is appropriate for the D2D scenario. Additionally, a well-known D2D coded caching converse was proposed in [8], and it has been demonstrated that, when the memory size is large, the proposed D2D caching and delivery scheme is order optimal within a constant factor. It is difficult to find the optimal caching and delivery scheme and the corresponding optimal rate–memory tradeoff for the centralized D2D coded caching problem. However, there are many researchers who try to find the fundamental limits of the centralized D2D coded caching problems under certain assumptions or additional constraints, e.g., [9,10].

With concern to the timeliness of the communication, *one-shot delivery*, which is defined to satisfy the condition that each user can decode any bit of its requested file from its own cache and the transmitted signal from at most one other user, is proposed in [9] for the centralized D2D coded caching problem. For example, one self-driving vehicle may quickly decode the requested map data after receiving the signals transmitted by another self-driving vehicle, without waiting for all the considered vehicles to complete the transmission of signals. The proposed caching and delivery scheme in [9] is optimal under the constraint of uncoded cache placement and one-shot delivery, and it is order optimal within a factor of two if the converse of the shared-link coded caching problem with uncoded cache placement [11] is used as the lower bound and order optimal within a factor of four compared to the general D2D coded caching converse results.

In addition to [9], many other researchers study variants of the D2D coded caching problems, such as allowing for coded placement with three users [10], private caching [12], private caching with a trusted server [13,14], distinct cache sizes [15], finite file packetizations [16], finite-length analysis [17], secure coded caching [18], secure delivery [19], wireless multi-hop D2D networks [20,21], partially cooperative D2D communication networks [22,23], constructions of placement delivery arrays (PDAs) [24], and so on. Among these papers, most of them assume that all users will request content at the beginning of the delivery phase. However, in practice, this may not be true. For example, when assisted self-driving vehicles within a certain range carry out D2D communication, they may not request at the same time or some of them may be driven manually and do not need to access high-definition map data. In these situations, waiting for all users to request content will waste time, and setting the requests of the users who do not request some arbitrary file demand will waste communication resources. Note that in these scenarios, even though the users may not request data, they are still available to participate in the delivery phase by transmitting signals that are functions of their cached contents.

Hence, in this paper, we propose and study a new problem called *request-robust D2D coded caching*, where in the delivery phase, though all users in the placement phase are still present and may help with the transmission, some of them do not request any files. It is not known in the placement phase the number or identity of the users who do not request files. This problem is not the same as the decentralized D2D coded caching problem [8], where users who leave or turn off during the delivery phase do not make file requests, nor do they participate in the delivery. Note that this problem is similar to the user inactivity problem in the D2D caching setting [9,22], where each user may independently have a probability of being inactive, i.e., they do not make a file request at the beginning of the delivery phase. However, in the request-robust D2D coded caching problem, inactive users still help in the delivery phase by transmitting signals, whereas in the user inactivity problem, they do not.

### 1.1. Main Contributions

The main contributions of the paper can be summarized as follows:

(1) For the request-robust D2D coded caching problem, we adapt the scheme from [9] for uncoded cache placement and one-shot delivery and call the adapted scheme the *adapted Yapar–Wan–Schaefer–Caire (YWSC) scheme*.
(2) In order to find better performance, we present a new achievable scheme based on the uncoded cache placement and exploiting common demands [11] and one-shot delivery [9]. The caching strategy is the same as that proposed by Maddah-Ali and Niesen in [3] (Algorithm 1), while the delivery strategy divides the sub-files into three categories, and different delivery signals are designed for each category. We call the new scheme the *three-category-based scheme*. This scheme was presented in the conference version of this paper [25].
(3) We propose an information-theoretic lower bound under uncoded cache placement based on seeking the converse of a problem called coded caching with inactive users. The problem of coded caching with inactive users was proposed in [26], where users are inactive with a certain probability in the traditional coded caching problem of [3]. Hence, the converse for the problem of coded caching with inactive users can serve as a converse for the request-robust D2D coded caching problem. Note that [26] only considers the optimization of the cache replication parameter and does not provide a converse for the caching and delivery scheme.
(4) We prove that the performance of the adapted YWSC scheme is order optimal within a factor of two under the assumption of uncoded cache placement and within a factor of four in general.
(5) Through numerical evaluation, we show that the three-category-based scheme outperforms the adapted YWSC scheme, as well as other known D2D coded caching schemes [3] applied to the request-robust scenario.

### 1.2. Notations

Throughout this paper, H(·) represents the entropy of random variables, |·| represents the cardinality of a set, ⊕ denotes finite field addition, we let $X\backslash Y\overset{▵}{=}\left\{x\in X|x\notin Y\right\}$, $\left[x:y:z\right]\overset{▵}{=}\left\{x,x+y,x+2y,...,z\right\}$, [x:y]=[x:1:y] and [n]=[1:n]. For two integers *x*, and *y*, if x<y or x≤0, we let yx=0.

## 2. System Model and Related Background

### 2.1. System Model

**Figure 1:**
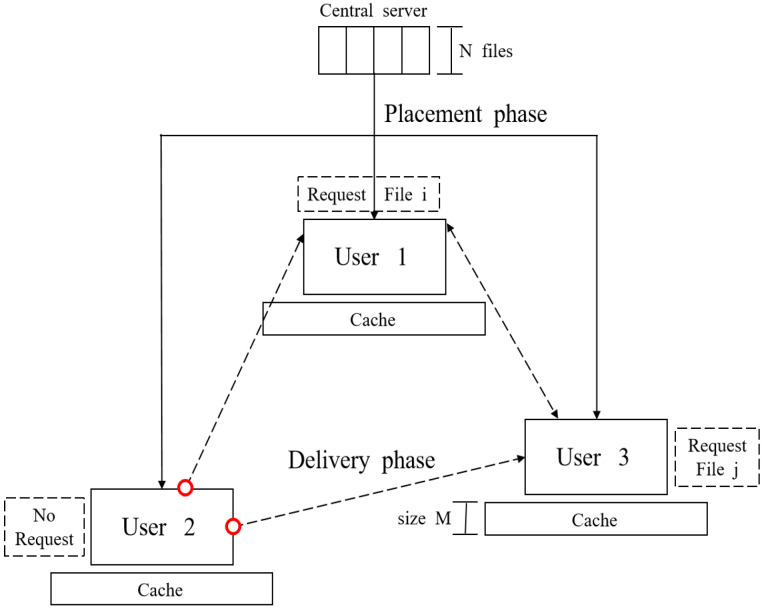
System model for request-robust D2D coded caching problem when there are 3 users. In this realization, User 2 does not request. Solid and dotted lines indicate placement and delivery phases, respectively.

We study the *request-robust D2D coded caching* problem, which is defined in the following. We consider a D2D coded caching system (see Figure 1) where a server is connected to a fixed content file database of *N* files, $W\overset{▵}{=}\left(W_{1},\ldots ,W_{N}\right)$. Each file consists of *F* bits. There are *K* users in the system, each with a cache of size MF bits. We focus on the non-trivial scenario where M≤N. Let K be the set of user indices, i.e., $K\overset{▵}{=}\left[K\right]$.

The system operates in two phases. In the placement phase, each user’s cache is filled by the central server, which does not know the number of users or the identities of the users requesting files in the other phase. Denote the content in the cache of User *k* as $Z_{k}$, k∈K. In the delivery phase, some of the *K* users will make file requests while others will not. We denote the set of users making file requests as R, R⊆K. Each user in R will request a single file. Let *r* denote the number of users requesting files, i.e., r≜|R|, and we assume that the file requests, i.e., which user requests which file and which users are not requesting any files, are known to all *K* users. Each of the *K* users will send a signal that will be received by the users in R. It is required that each user in R can decode its requested file by using the signals received and its own cache content. Note that Figure 1 is different from [9] (Figure 1), i.e., there exists a user who does not request any file in Figure 1, while in [9] (Figure 1), all users request files.

More specifically, a caching and delivery scheme for this system consists of

1. *K* caching functions
  $$\varphi_{k}:{\left[2^{F}\right]}^{N}\to \left[2^{MF}\right],\;k\in K,$$
  which map the *N* files into cache contents of the users, denoted by $Z_{k}=\varphi_{k}\left(W_{1},\ldots ,W_{N}\right),k\in K$. Thus, we have the following entropy constraint:
  $$\begin{array}{cc} H(Z_{k}|W_{1},W_{2},\ldots ,W_{N})=0,\; & k\in K. \end{array}$$
2. K∑r=1KKrNr encoding functions
  $$\phi_{k}^{D_{R}}:\left[2^{MF}\right]\to \left[2^{R_{k}F}\right],\;k\in K.$$
  where $D_{R}$ is the set of file requests made by the users in R. For example, if there are K=4 users, and Users 1 and 3 do not request files during the delivery phase, the request vector is $D_{\left\{2,4\right\}}=\left(d_{2},d_{4}\right)$. The encoding function $\phi_{k}^{D_{R}}$ denotes the mapping of User *k* from its cached content to the signal it transmits, which is denoted as $X_{k}^{D_{R}}$, i.e., $X_{k}^{D_{R}}\overset{▵}{=}\phi_{k}^{D_{R}}\left(Z_{k}\right)$. Thus, we have
  (1) $$\begin{array}{cc} H(X_{k}^{D_{R}}|Z_{k})=0,\; & k\in K. \end{array}$$
  We assume the signal $X_{k}^{D_{R}}$ consists of $R_{k}^{D_{R}}F$ bits. The signals transmitted by all *K* users consist of $R^{D_{R}}F$ bits, i.e., $R^{D_{R}}=\sum_{k=1}^{K}R_{k}^{D_{R}}$.
3. ∑r=1KKrrNr decoding functions
  $$\psi_{k}^{D_{R}}:\left[2^{MF}\right]\times \left[2^{F\sum_{u\in K\setminus \{k\}}R_{u}^{D_{R}}}\right]\to \left[2^{F}\right],\;k\in R,$$
  which is the decoding function used by User *k*. For example, if there are K=4 users, and Users 1 and 3 do not request any file during the delivery phase, the decoded files at Users 2 and 4 are ${\hat{W}}_{d_{2}}=\psi_{2}^{\left(d_{2},d_{4}\right)}\left(Z_{2},X_{1}^{\left(d_{2},d_{4}\right)},X_{3}^{\left(d_{2},d_{4}\right)},X_{4}^{\left(d_{2},d_{4}\right)}\right)$ and ${\hat{W}}_{d_{4}}=\psi_{4}^{\left(d_{2},d_{4}\right)}\left(Z_{4},X_{1}^{\left(d_{2},d_{4}\right)},X_{2}^{\left(d_{2},d_{4}\right)},X_{3}^{\left(d_{2},d_{4}\right)}\right)$, respectively.

Correct decoding by the users requesting files is given by ${\hat{W}}_{d_{k}}=W_{d_{k}},k\in R$, or in other words,

(2) $$\begin{array}{cc} H(W_{d_{k}}|Z_{k},X_{\left[K]\setminus \{k\right\}}^{D_{R}})=0,\; & k\in R, \end{array}$$

which is called the decodability constraint. We find that by combing (1) and (2), one can decode any file by knowing the cache of all users, i.e.,

(3) $$H(W_{\left[N\right]}|Z_{\left[K\right]})=0.$$

which implies that we are interested in the case where KM≥N.

For any caching and delivery scheme that satisfies the decodability constraint, for a fixed R with size *r*, we define $D^{R}$ as the set of all possible demands ${\left\{1,\cdots ,N\right\}}^{r}$. We are interested in two performance metrics: one is with respect to the average performance, and the other is with respect to the worst performance. More specifically, the average performance is defined as follows: we assume that the request vector $D_{R}$ is uniformly distributed on $D^{R}$. Then, the average delivery rate with respect to the uniform demand $R_{\mathrm{ave},\mathrm{req}-\mathrm{rob}}^{R}$ is defined as

$$R_{\mathrm{ave},\mathrm{req}-\mathrm{rob}}^{R}=E_{D_{R}}\left[R^{D_{R}}\right].$$

For a given *r*, we define the maximum average delivery rate with respect to the uniform demand $R_{\mathrm{ave},\mathrm{req}-\mathrm{rob}}^{r}$, where the maximum is over all request sets R with size *r*, i.e.,

$$\begin{array}{c} R_{\mathrm{ave},\mathrm{req}-\mathrm{rob}}^{r}=\max_{R:|R|=r}R_{\mathrm{ave},\mathrm{req}-\mathrm{rob}}^{R} \end{array}$$

The worst-case performance is defined as follows: first, the worst-case delivery rate $R_{\mathrm{worst},\mathrm{req}-\mathrm{rob}}^{R}$ is defined as

$$R_{\mathrm{worst},\mathrm{req}-\mathrm{rob}}^{R}=\max_{D_{R}}R^{D_{R}}.$$

For a given *r*, we define the maximum worst-case delivery rate $R_{\mathrm{worst},\mathrm{req}-\mathrm{rob}}^{r}$, where the maximum is over all request sets R with size *r*, i.e.,

$$R_{\mathrm{worst},\mathrm{req}-\mathrm{rob}}^{r}=\max_{R:|R|=r}R_{\mathrm{worst},\mathrm{req}-\mathrm{rob}}^{R}.$$

We would like to design caching and delivery schemes such that $R_{\mathrm{ave},\mathrm{req}-\mathrm{rob}}^{r}$ and $R_{\mathrm{worst},\mathrm{req}-\mathrm{rob}}^{r}$ are both the smallest for every r=1,2,⋯,K. As can be seen, this is a multi-objective optimization.

For easy presentability of the results, following the notation of [11], we denote $N_{e}\left(D_{R}\right)$ as the number of distinct files in a request vector $D_{R}$. $D_{R\setminus \{k\}}$ and $N_{e}\left(D_{R\setminus \{k\}}\right)$ are denoted as the request vector of users R∖{k} and the number of distinct files requested by all requesters but User *k*, respectively.

### 2.2. Preliminaries

In this subsection, we briefly summarize the related approaches, namely the uncoded symmetric placement scheme in [3] and the problem of coded caching with inactive users, which are critical for building our results for the request-robust D2D coded caching problem.

#### 2.2.1. Uncoded Symmetric Placement Scheme

First, we introduce the uncoded symmetric placement scheme, which is useful for our scheme proposed in Section 4.

**Definition** **1.** *(Maddah-Ali Niesen [MAN] Uncoded Symmetric Placement Scheme): Define t as t=KM/N. When t is an integer, we have the MAN uncoded symmetric placement scheme as follows: Each file $W_{n}$ is divided into Kt disjoint sub-files denoted by $W_{n,T}$, where n∈[N], T⊆K, |T|=t, and H(Wn,T)=F/Kt. Each user k caches all the bits of the sub-files $W_{n,T}$, n∈[N], for all T∋k. Since each file includes K−1t−1 sub-files with T∋k, each user k satisfies the memory constraint H(Zk)=NFK−1t−1H(Wn,T)=NFt/K=MF.*

For the convenience of understanding and reference, we give the algorithm of this scheme in Algorithm 1.
**Algorithm 1** MAN Uncoded Symmetric Placement Scheme $\left(N,K,M,W_{\left[N\right]}\right)$1:t←KM/N2:T←{𝒯⊆[K]:|𝒯|=t}3:**for** n∈N **do**4:   Divide file $W_{n}$ into disjoint sub-files ($W_{n,𝒯}:𝒯\in T$) with equal size5:**end for**6:**for** k∈[K] **do**7:   $Z_{k}\leftarrow (W_{n,𝒯}:n\in \left[N\right],𝒯\in T$, k∈𝒯)8:**end for**

The uncoded symmetric placement scheme is the optimal achievable placement scheme both for the shared-link model with uncoded cache placement [3] and D2D work with uncoded cache placement and one-shot delivery [9], which reveals that regardless of the number of users, using the uncoded symmetric placement scheme can satisfy the optimal rate of these models in all cases. Due to the superiority of the uncoded symmetric placement scheme, we use the scheme as the placement scheme in our scheme proposed in Section 4.

#### 2.2.2. Problem of Coded Caching with Inactive Users

We denote our original D2D model with *r* users of *K* users requesting files independently in W as System 1. In order to derive the converse of System 1, we consider another system model named *coded caching with inactive users*, denoted as System 2. This is the model where a central server responds to the users’ requests, and some of the users do not request any files in the delivery phase. The central server connects to the whole file database.

The placement phase of System 2 is exactly the same as that of System 1. Thus, in System 2, Equation (3) is still satisfied. The delivery phase of System 2 is different from that of System 1. Specifically, in System 1, the codewords are transmitted by the users, while in System 2, the codewords are transmitted by the central server. Since the central server has the whole database and connects to all *K* users, while in System 1, each user only caches a subset of the whole database and only connects to other K−1 users, the optimal maximum average and worst-case delivery rate in System 2, denoted as $R_{\mathrm{ave},\mathrm{inactive}}^{r*}$ and $R_{\mathrm{worst},\mathrm{inactive}}^{r*}$, can not be larger than the delivery rate in System 1, respectively. In other words, we have the following inequality:

(4) $$\begin{array}{cc} R_{\mathrm{ave},\mathrm{req}-\mathrm{rob}}^{r*} & \geq R_{\mathrm{ave},\mathrm{inactive}}^{r*}, \end{array}$$

(5) $$\begin{array}{cc} R_{\mathrm{worst},\mathrm{req}-\mathrm{rob}}^{r*} & \geq R_{\mathrm{worst},\mathrm{inactive}}^{r*}. \end{array}$$

## 3. Main Result

In this section, we present the main results of this work. We propose two achievable schemes for the request-robust D2D coded caching problem in Theorems 1 and 2. We further propose a converse for the problem of coded caching with inactive users in Theorem 3, which also serves as a converse result to the request-robust D2D coded caching problem. Theorem 4 compares the performance gap between the achievability result in Theorem 1 and the converse result in Theorem 3 and shows that they are within a multiplicative gap.

The first achievable scheme is obtained by adapting the achievable scheme in [9] with uncoded cache placement and one-shot delivery to the request-robust D2D coded caching problem. More specifically, the adaptation is performed by assigning the users, who do not request, a demand that is most requested by the requesters. We call the adapted scheme the *adapted Yapar–Wan–Schaefer–Caire (YWSC) scheme*. We denote the adapted request vector as $D_{K}^{\prime }$ and the adapted request vector of users K∖{k} as $D_{K\setminus \{k\}}^{\prime }$. Hence, we have $N_{e}\left(D_{K}^{\prime }\right)=N_{e}\left(D_{R}\right)$ and obtain Theorem 1 as follows:

**Theorem** **1.** *For the request-robust D2D coded caching problem, the optimal maximum average delivery rate with respect to the uniform demand is upper bounded by*

(6) Rave,req-robr*≤EDK′KK−1t−∑i=1KK−1−Ne(DK∖{i}′)t−fK−rt−K−r−1ttKt,

when $t=\frac{KM}{N}$ is an integer in [K], where f is an integer equal to one if and only if each requester demands a distinct file, i.e., $N_{e}\left(D_{R}\right)=r$; otherwise, f=0. When t∉[K], $R_{\mathrm{ave},\mathrm{req}-\mathrm{rob}}^{r*}$ is upper bounded by the lower convex envelope of the values in (6) for integer values of t∈[K].

*For the maximum worst-case delivery rate, we have*

(7) Rworst,req-robr*≤maxDK′KK−1t−∑i=1KK−1−Ne(DK∖{i}′)t−fK−rt−K−r−1ttKt,

(8) =KK−1t−(K−r)K−r−1t−rK−rttKt,r≤N,KK−1t−(2N−r)K−Nt−(K+r−2N)K−1−NttKt,otherwise,KK−1t−K−1−NttKt,r≥2N,

where $t=\frac{KM}{N}$ is an integer in [K]. When t∉[K], $R_{\mathrm{worst},\mathrm{req}-\mathrm{rob}}^{r*}$ is upper bounded by the lower convex envelope of the values in (7) for integer values of t∈[K].

**Proof.**  The proof of Theorem 1 is provided in Appendix A. □

Theorem 1 is a simple adaptation of an existing scheme; in order to improve its performance, we propose a new scheme, called the *three-category-based scheme*, and obtain Theorem 2 as follows:

**Theorem** **2.** *For the request-robust D2D coded caching problem, the optimal maximum average delivery rate with respect to the uniform demand is upper bounded by*

(9) Rave,req-robr*≤EDR{∑i=max{1,t+r−K}min{t−1,r−1}K−rt−iri+1−r−Ne(DR)i+1Kt+K−rtNe(DR)Kt+rr−1t−∑i∈Rr−1−Ne(DR∖{i})ttKt},

where $t=\frac{KM}{N}$ is an integer in [K]. When t∉[K], $R_{\mathrm{ave},\mathrm{req}-\mathrm{rob}}^{r*}$ is upper bounded by the lower convex envelope of the values in (9) for integer values of t∈[K].

*Then, for the maximum worst-case delivery rate, we have*

Rworst,req-robr*≤maxDR{∑i=max{1,t+r−K}min{t−1,r−1}K−rt−iri+1−r−Ne(DR)i+1Kt

(10) +K−rtNe(DR)Kt+rr−1t−∑i∈Rr−1−Ne(DR∖{i})ttKt},

(11) =∑i=max{1,t+r−K}min{t−1,r−1}K−rt−iri+1Kt+rK−rtKt+rr−1ttKt,r≤N,∑i=max{1,t+r−K}min{t−1,r−1}K−rt−iri+1−r−Ni+1Kt+NK−rtKt+rr−1t−(2N−r)r−Nt−2(r−N)r−1−NttKt,otherwise,∑i=max{1,t+r−K}min{t−1,r−1}K−rt−iri+1−r−Ni+1Kt+NK−rtKt+rr−1t−r−1−NttKt,r≥2N,

where $t=\frac{KM}{N}$ is an integer in [K]. When t∉[K], $R_{\mathrm{worst},\mathrm{req}-\mathrm{rob}}^{r*}$ is upper bounded by the lower convex envelope of the values in (10) for integer values of t∈[K].

**Proof.**  In proving Theorem 2, we propose a new scheme, i.e., the three-category-based scheme, where the sub-files are divided into three categories and different delivery signals are designed for each category. The detailed proof can be found in Section 4. □

**Remark** **1.** *In Section 5, we numerically compare the delivery rates of the three-category-based scheme and the adapted YWSC scheme, and it can be seen that the proposed three-category-based scheme outperforms the adapted YWSC scheme in all cases cited (see Section 5 for the cited cases and comparison results).*

In the following theorem, we characterize a converse for the request-robust D2D coded caching problem.

**Theorem** **3.** *For the request-robust D2D coded caching problem, the optimal maximum average delivery rate with respect to the uniform demand and under the constraint of uncoded placement is lower bounded by:*

(12) Rave,req-robr*≥EDRKt+1−K−Ne(DR)t+1Kt,

where $t=\frac{KM}{N}$ is an integer in [K], where $D_{R}$ is uniformly distributed over $D^{R}$. When t∉[K], $R_{\mathrm{ave},\mathrm{req}-\mathrm{rob}}^{r*}$ is lower bounded by the lower convex envelope of the values in (12) for integer values of t∈[K].

*Then, for the maximum worst-case delivery rate, we have that the optimal maximum worst-case delivery rate under the constraint of uncoded placement is lower bounded by*

(13) Rworst,req-robr*≥Kt+1−K−min{r,N}t+1Kt,

where $t=\frac{KM}{N}$ is an integer in [K]. When t∉[K], $R_{\mathrm{worst},\mathrm{req}-\mathrm{rob}}^{r*}$ is lower bounded by the lower convex envelope of the values in (13) for integer values of t∈[K].

**Proof.**  Similar to [9], we use the converse of the central server version, i.e., System 2, as a converse for the request-robust D2D coded caching problem when the converse is under the constraint of uncoded placement. The detailed proof is provided in Appendix B. □

We compare the rate achieved by the adapted YWSC scheme from Theorem 1 and the converse present in Theorem 3 and obtain a multiplicative gap result of Theorem 4 as follows:

**Theorem** **4.** *For the request-robust D2D coded caching problem, the upper bounds of the optimal maximum average and worst-case rates from Theorem 1 are order optimal within a factor of two under the constraint of uncoded cache placement and within a factor of four in general.*

**Proof.**  The proof of Theorem 4 is given in Appendix D. □

**Remark** **2.** *It is hard to analytically prove that the rate achieved by the three-category-based scheme from Theorem 2 outperforms the rate achieved by the adapted YWSC scheme from Theorem 1 in all cases. However, since in the numerical comparisons in Section 5 the three-category-based scheme performs better than the adapted YWSC scheme in all cases cited, we conjecture that the rate achieved by the three-category-based scheme from Theorem 2 and the converse present in Theorem 3 also follow the multiplicative gap characterized by Theorem 4.*

## 4. A Novel Achievable Scheme, i.e., Proof of Theorem 2

In this section, we present an achievable scheme for the request-robust D2D coded caching problem. The scheme achieves the rate stated in Theorem 2. We will first provide a general achievable scheme, which is based on uncoded cache placement and exploiting common demands [11] and one-shot delivery [9]. Then, we will characterize the performance of the proposed scheme and show that for any requester set R and corresponding request vector $D_{R}$, the proposed three-category-based scheme achieves the rate

(14) Rreq-robDR=∑i=max{1,t+r−K}min{t−1,r−1}K−rt−iri+1−r−Ne(DR)i+1Kt+K−rtNe(DR)Kt+rr−1t−∑i∈Rr−1−Ne(DR∖{i})ttKt,

which, with the explicit characterization of the maximum worst-case delivery rate in Section 4.2, immediately proves Theorem 2. Finally, we will provide an example to aid in a better understanding of the proposed three-category-based scheme.

### 4.1. General Scheme

For the placement phase, because the central server does not know the number of requesters *r*, we use the highly adaptable MAN uncoded symmetric placement scheme described in Definition 1, denoted as $M_{\mathrm{MAN}}$. In the following, we restrict to integer values of t∈[1:K]. For cache size *M* where t=KM/N is not an integer, memory-sharing will be performed [3,8].

For the delivery phase, let the set of requesters be R with size *r*. The *r* requesters each demand a single file. The delivery strategy is divided into the following steps:

(a) **Determining the leading requesters**: Each user k∈K∖R who does not request arbitrarily selects a subset of $N_{e}\left(D_{R}\right)$ requesters, denoted by $U^{\prime k}=\left\{u_{1}^{\prime k},...,u_{N_{e}\left(D_{R}\right)}^{\prime k}\right\}\subseteq R$, that request $N_{e}\left(D_{R}\right)$ distinct files. Using the idea of leaders from [11], we name these requesters as the *leading requesters of User k*.
(b) **Splitting the sub-files into three categories**: Recall that each sub-file is denoted as $W_{n,𝒯}$ and is cached by only users in 𝒯. If 𝒯⊆K∖R, then this sub-file belongs to the first category, which is the set of sub-files that are only cached by users who do not request any files. If 𝒯 contains some elements from R and some elements from K∖R, then this sub-file belongs to the second category, which is the set of sub-files that are cached by both requesters and non-requesters. Finally, if 𝒯⊆R, then this sub-file belongs to the third category, which is the set of sub-files that are only cached by users who make file requests.

The three categories may not all exist or be required by requesters, and it depends on the value of *r* and *t*. When t∈[K−r+1,K] or r=K, the first category does not exist. When t=1 or r=K, the second category does not exist, and when r=1 or t=K, the second category is not required. When t∈[r+1,K], the third category does not exist, and when t=r or r=1, the third category is not required.

(c) **Transmitting signals for the sub-files in the three categories**: we will discuss the delivery scheme for the sub-files in each of the three categories.

(i) For the sub-files in the first category needed by Requester k,k∈R, since these sub-files are not cached in any of the requesters, the users in K∖R who cache these sub-files transmit them in an uncoded form. Suppose $W_{d_{k},𝒯}$ is requested by User k∈R, 𝒯⊆K∖R, any of the *t* users in 𝒯 can transmit the sub-file in an uncoded form. However, we adopt the file-splitting strategy in [9] and allow each user in 𝒯 to transmit 1/t part of the sub-file, i.e., $W_{d_{k},𝒯}$ is divided into *t* pieces, each consisting of FtKt numbers of bits. The pieces are denoted as $W_{d_{k},𝒯,a}$, a∈𝒯, and User *a* transmits
  (15) $$X_{a}^{1\mathrm{st},d_{k},𝒯}=W_{d_{k},𝒯,a}.$$
  We notice that $W_{d_{k},𝒯,a}$ may be needed by other requesters, i.e., there may be other requesters that request file $d_{k}$ also. Hence, we let each user *a* transmit in sequence $X_{a}^{1\mathrm{st},d_{k},𝒯}$ for all $k\in U^{\prime a}$. Hence, the rate of transmitting total bits for the sub-files in the first category is
  (16) R1st=tK−rtNe(DR)tKt=K−rtNe(DR)Kt,
  because for each 𝒯, every user a∈𝒯 transmits Ne(DR)FtKt bits, and there are *t* users in each 𝒯, and a total of K−rt number of 𝒯 that are subsets of K∖R.
(ii) Consider a sub-file in the second category needed by Requester k,k∈R, denoted as $W_{d_{k},𝒯}$, where k∉𝒯. Denote the set of elements in 𝒯 that are in R as B, whose size is denoted as *i*, and we have 1≤i≤r−1, because we know at least User *k* who is requesting a file is not in 𝒯. Further denote the set of elements of 𝒯 that are in K∖R as $\hat{B}$, whose size is t−i, and we have 1≤t−i≤K−r. Hence, 𝒯 can be written as $𝒯=B⋃\hat{B}$. Furthermore, *i* must satisfy i∈[max{1,t+r−K},min{t−1,r−1}].

Consider the set $\bar{B}≜\left\{k\right\}⋃B$, which is a set consisting of i+1 requesters. The sub-file $W_{d_{x},\hat{B}⋃\bar{B}\setminus \left\{x\right\}}$ is needed for $x\in \bar{B}$. We ask that this sub-file be transmitted by the t−i non-requesters, and $W_{d_{x},\hat{B}⋃\bar{B}\setminus \left\{x\right\}}$ for any x∈B be divided into t−i equal-length disjoint sub-pieces of F(t−i)Kt bits, which are denoted by $W_{d_{x},\hat{B}⋃\bar{B}\setminus \left\{x\right\},b}$, where $b\in \hat{B}$. Hence, if User $b\in \hat{B}$ transmits

(17) $$X_{b}^{2\mathrm{nd},d_{x},\hat{B}⋃\bar{B}\setminus \left\{x\right\}}=\underset{x\in \bar{B}}{⨁}W_{d_{x},\hat{B}⋃\bar{B}\setminus \left\{x\right\},b},$$

the sub-piece retrieval can be accomplished for each requester in $\bar{B}$ since User *x* has all the sub-pieces on the RHS of (17), except for $W_{d_{x},\hat{B}⋃\bar{B}\setminus \left\{x\right\},b}$.

We ask each user b∈K∖R to transmit $X_{b}^{2\mathrm{nd},d_{x},\hat{B}⋃\bar{B}\setminus \left\{x\right\}}$ in sequence, only if $\bar{B}\cap U^{\prime b}\neq \emptyset$, i.e., user *b* will not transmit if the set $\bar{B}$ consists of only non-leading requesters. We now count the amount of transmission for the second category. For a fixed *i*, the number of $\hat{B}$, which is of size t−i, is K−rt−i. For each $u\in \hat{B}$, the number of transmitted bits is ri+1−r−Ne(DR)i+1 times the size of a sub-piece, and there are a total of t−i users in $\hat{B}$. Hence, the rate of transmitting all the bits for the sub-files in the second category is

(18) R2nd=∑i=max{1,t+r−K}min{t−1,r−1}K−rt−iri+1−r−Ne(DR)i+1Kt.

The next lemma shows that the scheme proposed satisfies the decodability constraint, even for the non-leading requesters.

**Lemma** **1.** *The scheme proposed for the sub-files in the second category satisfies the decodability constraint, i.e., (2).*

The proof of Lemma 1 is based on showing the equivalence of the designed scheme and that in [11]. Hence, we state the following remark first:

**Remark** **3.** *Notice that when the sub-files are in the second category and the parameter t is fixed, for each partition integer i, a user $b\in \hat{B}$ generates its codewords exclusively from the sub-pieces $W_{d_{k},\hat{B}\cup B,b}$, and there exist ri such sub-pieces in its cache. In addition, for any $c\in \hat{B}\setminus \left\{b\right\}$, we have $W_{d_{k},\hat{B}\cup V^{b},b}\cap W_{d_{l},\hat{B}\cup V^{c},c}=\emptyset$ for any $V^{b},V^{c}\subseteq R,|V^{b}\left|=\right|V^{c}|=i,k\in V^{b},l\in V^{c}$. That is to say, users in $\hat{B}$ generate their codewords based on non-overlapping libraries of size (t−i)NriF(t−i)Kt=NriF/Kt bits. Also, observe that the cache of requester k contains (t−i)r−1i−1 such $W_{d_{k},\hat{B}\cup V^{b},b}$ sub-pieces, which amount to N(t−i)r−1i−1F(t−i)Kt=Nr−1i−1FKt=NirtFrKt bits.*

*Therefore, the proposed scheme is in fact composed of (t−i) shared-link models [3] each with N files of size F′=riF/Kt bits and $K^{\prime }=r$ users with caches of size $M^{\prime }=Ni/r$ units each. The corresponding parameter for each model is found to be $t^{\prime }=\frac{K^{\prime }M^{\prime }}{N}=i$. To ensure the existence of sub-files in the second category, the partition integer must satisfy i∈[max{1,t+r−K},min{t−1,r−1}]. Hence, for every i∈[max{1,t+r−K},min{t−1,r−1}], summing the achievable rates $R_{sl}$, which is defined as follows from [11]:*

(19) Rsl=Kt+1−K−Ne(d)t+1Kt,

*of each $b\in \hat{B}$ shared-link sub-system and replacing the shared-link system parameters $F,K,M,t,and\;N_{e}\left(d\right)$ with $F^{\prime },K^{\prime },M^{\prime },t^{\prime },and\;N_{e}\left(D_{R}\right)$, respectively, we obtain (18).*

We now prove that for each partition integer i∈[max{1,t+r−K},min{t−1,r−1}], each requester *k* is able to decode the needed sub-files in the second category with the partition integer *i* upon receiving the codewords $X_{b}^{2\mathrm{nd},d_{k},\hat{B}⋃\bar{B}\setminus \left\{k\right\}}$ for all $b\in \hat{B}$.

When *k* is a leading requester of the user *b* who does not request, i.e., $k\in U^{\prime b}$, it can decode any required sub-piece $W_{d_{k},\hat{B}\cup P^{k},b}$, where $P^{k}\subseteq R\setminus \left\{k\right\},\left|P^{k}\right|=i$, from $X_{b}^{2\mathrm{nd},d_{k},\hat{B}⋃P^{k}}$, which is broadcast from user *b* by performing

$$W_{d_{k},\hat{B}\cup P^{k},b}=\left(\underset{x\in P^{k}}{⨁}W_{d_{x},\hat{B}\cup P^{k}\cup \left\{k\right\}\setminus \left\{x\right\},b}\right)⨁X_{b}^{2\mathrm{nd},d_{k},\hat{B}⋃P^{k}},$$

as can be seen from (17).

When $k\notin U^{\prime b}$, it is less straightforward for the non-leading requester *k* to decode the needed sub-files, because not all of the corresponding codewords $X_{b}^{2\mathrm{nd},d_{k},\hat{B}⋃P^{k}}$ for its required sub-pieces $W_{d_{k},\hat{B}\cup P^{k},b}$ are directly broadcast from user *b*. However, Requester *k* can generate these codewords simply based on the codewords received. To show this, we reformulate the following Lemma 2 from [11] (Lemma 1), which is applied to the codewords broadcast by the user *b* with the partition integer *i*.

**Lemma** **2.** *Given an integer t, a partition integer i, a subset $\hat{B}\subseteq K\setminus R$ of size t−i, a user $b\in \hat{B}$, and a set of leading requesters $U^{\prime b}$, for any subset $C^{b}\subseteq R$ that includes $U^{\prime b}$, let $V_{F}^{b}$ be family of all subsets $V^{b}$ of $C^{b}$ such that each requested file in $D_{R}$ is requested by exactly one user in $V^{b}$. The following equation holds:*

(20) $$\underset{V^{b}\in V_{F}^{b}}{⨁}X_{b}^{2\mathrm{nd},d_{k},\hat{B}⋃\left\{C^{b}\setminus V^{b}\right\}\setminus \left\{k\right\}}=0,$$

*if each $X_{b}^{2\mathrm{nd},d_{k},\hat{B}⋃\left\{C^{b}\setminus V^{b}\right\}\setminus \left\{k\right\}}$ is defined in (17).*

**Proof.**  As we mentioned in Remark 3, for the sub-files needed in the second category, when the parameters *t* and *i* are fixed, the proposed scheme, in fact, corresponds to (t−i) shared-link schemes. Thus, Ref. [11] (Lemma 1) can directly be applied to each *b*-th shared-link scheme. □

Let us now consider any subset $\bar{B}$ of i+1 non-leading requesters of user *b* such that $\bar{B}\cap U^{\prime b}=\emptyset$. Using (20), the following equation can be derived:

(21) $$X_{b}^{2\mathrm{nd},d_{k},\hat{B}⋃\bar{B}\setminus \left\{k\right\}}=\underset{V^{b}\in V_{F}^{b}\setminus \left\{U^{\prime b}\right\}}{⨁}X_{b}^{2\mathrm{nd},d_{k},\hat{B}⋃\left\{C^{b}\setminus V^{b}\right\}\setminus \left\{k\right\}},$$

where $C^{b}=B^{\prime i}\cup U^{\prime b}$. Equation (21) shows that the codeword $X_{b}^{2\mathrm{nd},d_{k},\hat{B}⋃\bar{B}\setminus \left\{k\right\}}$ can be directly computed from the broadcast codewords transmitted to all the leading requesters of *b*, because all codewords on the RHS of (21) are directly broadcasted by user *b*. Hence, each requester *k* can obtain the value $X_{b}^{2\mathrm{nd},d_{k},\hat{B}⋃\bar{B}\setminus \left\{k\right\}}$ for any subset $\bar{B}$ of i+1 requesters and can decode its demanded sub-pieces as discussed before. Hence, Lemma 1 is proved.

(iii) Lastly, we consider the sub-files in the third category. Since all the sub-files needed for delivery are only cached in the requesters, the transmission will happen only among requesters. This is equivalent to the D2D coded caching model considered in [9], and we adopt its achievable scheme with uncoded cache placement and one-shot delivery, which we call the *Yapar–Wan–Schaefer–Caire (YWSC) scheme*.

More specifically, during the delivery phase, each sub-file is divided into *t* equal-length disjoint sub-pieces of FtKt bits, which are denoted by $W_{n,𝒯,i},i\in 𝒯$. Further, each user *i* in requester set R, where |R|=r, selects an arbitrary subset of $N_{e}\left(D_{R\setminus \{i\}}\right)$ users from R∖{i}, denoted by $U^{i}=\left\{u_{1}^{i},...,u_{N_{e}\left(D_{R\setminus \{i\}}\right)}^{i}\right\}$, which request $N_{e}\left(D_{R\setminus \{i\}}\right))$ distinct files and are referred to as *leading demanders of user i*. Then, for all subsets $E^{i}\subseteq R\setminus \left\{i\right\}$ of *t* users, each user *i* transmits

(22) $$\begin{array}{cc} \; & X_{i}^{3\mathrm{rd}}={\left\{Y_{E^{i}}^{i}\right\}}_{E^{i}\cap U^{i}\neq \emptyset }, \end{array}$$

where

(23) $$\begin{array}{cc} \; & Y_{E^{i}}^{i}=\underset{k\in E^{i}}{⨁}W_{d_{k},\left\{E^{i}\cup \left\{i\right\}\right\}\setminus \left\{k\right\},i}. \end{array}$$

In other words, since all users $k\in E^{i}$ shall retrieve the needed sub-pieces $W_{d_{k},\left\{E^{i}\cup \left\{i\right\}\right\}\setminus \left\{k\right\},i}$ from the transmissions of User *i*, using the idea of leaders from [11], each user *i* only needs to transmit in sequence the codewords $Y_{E^{i}}^{i}$ for all subsets $E^{i}$ such that $E^{i}\cap U^{i}\neq \emptyset$, i.e., $X_{i}^{3\mathrm{rd}}$.

As a result, when User *k* is a leading demander for User *i*, i.e., $k\in U^{i}$, it can decode any needed sub-piece $W_{d_{k},G^{k}\cup \left\{i\right\},i}$, where $G^{k}\subseteq R\setminus \left\{i,k\right\}$, $|G^{k}|=t-1$, from $Y_{G^{k}\cup \left\{k\right\}}^{i}$, which is transmitted from User *i* by performing

$$W_{d_{k},G^{k}\cup \left\{i\right\},i}=\left(\underset{x\in G^{k}}{⨁}W_{d_{x},\left\{G^{k}\cup \left\{i,k\right\}\right\}\setminus \left\{x\right\},i}\right)⨁Y_{G^{k}\cup \left\{k\right\}}^{i}.$$

When User *k* is not a leading demander for User *i*, using the equation

$$\underset{V^{i}\in V_{F}^{i}}{⨁}Y_{C^{i}\setminus V^{i}}^{i}=0,$$

proved in [9] (Lemma 1), where subset $C^{i}\subseteq R\setminus \left\{i\right\}$ includes $U^{i}$ and $V_{F}^{i}$ denotes the family of all subsets $V^{i}$ of $C^{i}$ such that each requested file in $D_{R\setminus \{i\}}$ is requested by exactly one user in $V^{i}$, each user *k* can decode its requested sub-piece through obtaining the value $Y_{E^{i}}^{i}$, for any subset $E^{i}$ of *t* users such that $E^{i}\cap U^{i}=\emptyset$, from the broadcast codewords by the following equation:

(24) $$Y_{E^{i}}^{i}=\underset{V^{i}\in V_{F}^{i}\setminus \left\{U^{i}\right\}}{⨁}Y_{C^{i}\setminus V^{i}}^{i},$$

where $C^{i}=E^{i}\cup U^{i}$. To sum up, for each i∈R∖{k}, User *k* decodes its requested sub-pieces by following either one of the strategies above, depending on whether it is a leading demander of User *i* or not.

For each user i∈R, the size of the transmitted signal amounts to r−1t−r−1−Ne(DR∖{i})t times the size of a sub-piece. Hence, the rate of transmitting all the bits for the sub-files in the third category is

(25) R3rd=rr−1t−∑i∈Rr−1−Ne(DR∖{i})ttKt,

In other words, the equivalence of the above scheme and that of [9] can be seen in the following remark:

**Remark** **4.** *For the sub-files in the third category, the proposed scheme is equivalent to that of [9]. The central server has a library of N files, $\left\{W_{n,𝒯}|𝒯\subset R,\left|𝒯\right|=t\right\}$, and each file has F˜≜rtFKt bits. There are r D2D users in the system, each requesting a single file. Based on the MAN uncoded symmetric placement scheme adopted, the placement is the same as [9], where each sub-file has the size of FKt, which is equal to F˜rt, and sub-file $W_{n,𝒯}$ is placed in users in 𝒯. Thus, each user caches a total of Nr−1t−1 sub-files. Hence, each user has a memory of*

M˜=Nr−1t−1FKt

*bits, and we can check that*

$$\begin{array}{c} \frac{r\tilde{M}}{N\tilde{F}}=t, \end{array}$$

*just as in [9]. Hence, for the sub-files in the third category, the equivalence between the proposed cache placement scheme and that of [9] is established. In other words, corresponding to the parameters (F,N,K,MF,t,andNe(d)) in [9], which denote the file size, number of files, number of users, cache size of each user, the parameter defined as $t=\frac{KMF}{NF}$, and the number of users requesting different files, respectively, we have $\left(\tilde{F},N,r,\tilde{M},t,and\;Ne\left(D_{R}\right)\right)$ in our problem.*

Summing up the three sub-schemes mentioned above, every requester can decode its requested file. Meanwhile, combining the rate from (16), (18), and (25), the total rate of this delivery scheme is $R_{\mathrm{req}-\mathrm{rob}}^{D_{R}}=R^{1\mathrm{st}}+R^{2\mathrm{nd}}+R^{3\mathrm{rd}}$, which results in the rate stated in (14). The description of this subsection with the explicit characterization of the maximum worst-case delivery rate in Section 4.2, directly proves Theorem 2. More specifically, since the proposed scheme is symmetric with respect to the users, any R with |R|=r will offer the same average delivery rate $R_{\mathrm{ave},\mathrm{req}-\mathrm{rob}}^{R}$ and the same worst-case delivery rate $R_{\mathrm{worst},\mathrm{req}-\mathrm{rob}}^{R}$.

**Remark** **5.** *The difference in transmitting the sub-files in the three categories is that the transmissions of the sub-files in the first and third categories both adopt the one-shot delivery scheme in [9], while the transmission of the sub-files in the second category adopts the common demands scheme in [11]. Moreover, the transmission of the sub-files in the first category is in uncoded form, while the transmissions of the sub-files in the second and third categories are both with coded multi-casting opportunity.*

We formally write the three-category-based scheme in Algorithm 2.

**Algorithm 2** Three-category-based Scheme (N,K,M)
**procedure** 
PLACEMENT($W_{1},\ldots ,W_{N}$)  1:Apply Algorithm 1 MAN Uncoded Symmetric Placement Scheme ($N,K,M,W_{\left[N\right]}$)**end procedure** 
 **procedure** 
DELIVERY($R,D_{R}$)  2:

$r\leftarrow |D_{R}|$

  3:

t←KM/N

  4:$N_{e}\left(D_{R}\right)\leftarrow$ the number of distinct elements in $D_{R}$  5:**for** k∈[K]∖R **do**  6: 
$U^{\prime k}\leftarrow \left\{u_{1}^{\prime k},\ldots ,u_{N_{e}\left(D_{R}\right)}^{\prime k}\right\}$  7:
**end for**
  8:(i) For sub-files in the first category:  9:

T←{𝒯⊆[K]∖R:|𝒯|=t}

10:**for** 𝒯∈T **do**11: **for** n∈[N] **do**12:  Divide sub-file $W_{n,𝒯}$ into *t* disjoint sub-pieces ($W_{n,𝒯,a}:a\in 𝒯$) with equal size13: **end for**14: **for** a∈𝒯 **do**15:  **for** $s\in U^{\prime a}$ **do**16:   User *a* transmit $X_{a}^{1\mathrm{st},d_{s},𝒯}=W_{d_{s},𝒯,a}$17:  **end for**18: **end for**19:
**end for**
20:(ii) For sub-files in the second category:21:**for** ∈[max{1,t+r−K},min{t−1,r−1}] **do**22: Ɓ $\leftarrow \{\hat{B}\subseteq \left[K\right]\setminus R:|\hat{B}|=t-i\}$23: **for** $\hat{B}\in$ Ɓ **do**24:  **for** B⊂R:|B|=i **do**25:   **for** n∈[N] **do**26:    Divide sub-file $W_{n,B\cup \hat{B}}$ into t−i disjoint sub-pieces ($W_{n,B\cup \hat{B},b}:b\in \hat{B}$) with equal size27:   **end for**28:  **end for**29:  **for** $b\in \hat{B}$ **do**30:   **for** $\bar{B}\subseteq R:\left|\bar{B}\right|=i+1$ **do**31:    **if** $\bar{B}\cap U^{\prime b}==\emptyset$ **then**32:     continue33:    **else**34:     User *b* transmits $X_{b}^{2\mathrm{nd},d_{x},\hat{B}⋃\bar{B}\setminus \left\{x\right\}}=\underset{x\in \bar{B}}{⨁}W_{d_{x},\hat{B}\cup \bar{B}\setminus \left\{x\right\},b}$35:    **end if**36:   **end for**37:  **end for**38: **end for**39:
**end for**
40:(iii) For sub-files in the third category:41:𝒢 ←{G⊆[K]:|G|=t}42:**for** n∈[N] **do**43: **for** G∈ 𝒢 **do**44:  Divide sub-file $W_{n,G}$ into *t* disjoint sub-pieces $\left(W_{n,G,i}:i\in G\right)$ with equal size45: **end for**46:
**end for**
47:**for** i∈R **do**48: $N_{e}\left(D_{R\setminus \{i\}}\right)\leftarrow$ the number of distinct elements in $D_{R\setminus \{i\}}$49: 
$U^{i}\leftarrow \left\{u_{1}^{i},...,u_{N_{e}\left(D_{R\setminus \{i\}}\right)}^{i}\right\}$50: **for** $E^{i}\subseteq R\setminus \left\{i\right\}:\left|E^{i}\right|=t$ users **do**51:  **if** $E^{i}\cap U^{i}==\emptyset$ **then**52:   continue53:  **else**54:   User *i* transmits $Y_{E^{i}}^{i}=\underset{k\in E^{i}}{⨁}W_{d_{k},\left\{E^{i}\cup \left\{i\right\}\right\}\setminus \left\{k\right\},i}$55:  **end if**56: **end for**57:
**end for**
**end procedure** 
 


### 4.2. The Maximum Worst-Case Delivery Rate

In this subsection, we characterize the performance of the proposed three-category-based scheme for the maximum worst-case delivery rate. The characterization is based on the observation that the binomial coefficient nm demonstrates a strictly ascending pattern with respect to *n*.

For the request-robust D2D coded caching problem, when N,K,M,r,andR do not change, since the upper bound rate $R_{\mathrm{req}-\mathrm{rob}}^{D_{R}}$ from (14) decreases as $N_{e}\left(D_{R}\right)$ decreases, the upper bound rate for the maximum worst-case delivery rate is the one at the maximum value of $N_{e}\left(D_{R}\right)$, i.e.,

(26) $$\begin{array}{c} N_{e}\left(D_{R}\right)=\min\left\{r,N\right\}. \end{array}$$

Then, for r≥2N, each file can be requested by at least two requesters, which leads to $N_{e}\left(D_{R\setminus \{i\}}\right),\forall i\in R$ having the maximum value of *N*. Hence, this case maximizes the upper bound rate $R_{\mathrm{req}-\mathrm{rob}}^{D_{R}}$.

For r<2N, a requester *i* may be the only user requesting a file or not, which leads to $N_{e}\left(D_{R\setminus \{i\}}\right)=N_{e}\left(D_{R}\right)-1$ and $N_{e}\left(D_{R\setminus \{i\}}\right)=N_{e}\left(D_{R}\right)$, respectively. Hence, due to (26), for r≤N, we have r−1−Ne(DR∖{i})t=0, which proves the case where r≤N.

For N<r<2N, we have $N_{e}\left(D_{R}\right)=N$ and then obtain that each file cannot be requested by more than two requesters. Thus, due to a total of *r* requesters, there are 2N−r requesters, each of which are the only users requesting a file while each of the remaining 2(r−N) requesters are not. Thus, we prove the case where N<r<2N.

### 4.3. Example

To aid in better understanding, we provide an example to illustrate the proposed scheme in Section 4.1.

Let us consider a case where N=2,K=6,andM=2/3. Hence, t=KM/N=2. In the placement phase, each file is divided into 62=15 sub-files, and each sub-file’s index is in T, where T is the family of all sets 𝒯 such that 𝒯⊂[6],|𝒯|=2. The user k∈[6] caches the following sub-files for each n∈{1,2}:

$$Z_{k}=\left\{W_{n,𝒯}|𝒯\in T,k\in 𝒯\right\}.$$

In the delivery phase, without a loss of generality, we consider only Users 1,2,3,and4 as requesters, each requesting a single file, i.e., R={1,2,3,4}, and the request vector is $D_{\left\{1,2,3,4\right\}}=\left(1,2,1,1\right)$. Notice that r=4 and $N_{e}\left(D_{\left\{1,2,3,4\right\}}\right)=2$. Requesters 1,2,3,and4 need the following missing sub-files:

$$\begin{array}{c} W_{1}\setminus Z_{1}=\left\{W_{1,\{2,3\}},W_{1,\{2,4\}},W_{1,\{2,5\}},W_{1,\{2,6\}},W_{1,\{3,4\}},W_{1,\{3,5\}},W_{1,\{3,6\}},W_{1,\{4,5\}},W_{1,\{4,6\}},W_{1,\{5,6\}}\right\},\\ W_{2}\setminus Z_{2}=\left\{W_{2,\{1,3\}},W_{2,\{1,4\}},W_{2,\{1,5\}},W_{2,\{1,6\}},W_{2,\{3,4\}},W_{2,\{3,5\}},W_{2,\{3,6\}},W_{2,\{4,5\}},W_{2,\{4,6\}},W_{2,\{5,6\}}\right\},\\ W_{1}\setminus Z_{3}=\left\{W_{1,\{1,2\}},W_{1,\{1,4\}},W_{1,\{1,5\}},W_{1,\{1,6\}},W_{1,\{2,4\}},W_{1,\{2,5\}},W_{1,\{2,6\}},W_{1,\{4,5\}},W_{1,\{4,6\}},W_{1,\{5,6\}}\right\},\\ W_{1}\setminus Z_{4}=\left\{W_{1,\{1,2\}},W_{1,\{1,3\}},W_{1,\{1,5\}},W_{1,\{1,6\}},W_{1,\{2,3\}},W_{1,\{2,5\}},W_{1,\{2,6\}},W_{1,\{3,5\}},W_{1,\{3,6\}},W_{1,\{5,6\}}\right\}. \end{array}$$

In Step (a), determining the leading requesters without a loss of generality, we assume that User 5 picks Users 1and2 and User 6 picks Users 2and3 as the leading requesters, i.e., $U^{\prime 5}=\left\{1,2\right\},U^{\prime 6}=\left\{2,3\right\}$.

In Step (b), we split the sub-files into three categories. More specifically, $W_{n,\{5,6\}}$, n∈[N] belong to the first category; $W_{n,\{1,5\}}$, $W_{n,\{1,6\}}$, $W_{n,\{2,5\}}$, $W_{n,\{2,6\}}$, $W_{n,\{3,5\}}$, $W_{n,\{3,6\}}$, $W_{n,\{4,5\}}$, $W_{n,\{4,6\}}$, n∈[N] belong to the second category; and $W_{n,\{1,2\}}$, $W_{n,\{1,3\}}$, $W_{n,\{1,4\}}$, $W_{n,\{2,3\}}$, $W_{n,\{2,4\}}$, $W_{n,\{3,4\}}$ belong to the third category.

In Step (c) of delivering signals, for the sub-files belonging to the first category, i.e., sub-files only cached by Users 5 and 6, after splitting these sub-files into two equal-length sub-pieces, from (15), we know that User 5 transmits

$$\begin{array}{c} W_{1,\{5,6\},5},W_{2,\{5,6\},5}, \end{array}$$

and User 6 transmits

$$\begin{array}{c} W_{1,\{5,6\},6},W_{2,\{5,6\},6}, \end{array}$$

which are all directly needed by the requesters. The rate $R^{1\mathrm{st}}=1/30\times 2\times 2=2/15$, which coincides with (16).

For the sub-files in the second category, since *i* must satisfy i∈[max{1,t+r−K},min{t−1,r−1}], in this example, we only need to consider i=1, which means that t−i=1. The fact that t−i=1 means that none of these sub-files need to be further split. Take, for example, User 1 in R; it requires the sub-file $W_{1,\{2,5\}}$. Thus, $𝒯=\left\{2,5\right\}=B⋃\hat{B}$, where B={2} and $\hat{B}=\left\{5\right\}$. Consider the set $\bar{B}=\left\{1\right\}⋃B=\left\{1,2\right\}$ of i+1=2 users. Following from (17), if User {5} transmits

$$\begin{array}{c} W_{1,\{2,5\}}⊕W_{2,\{1,5\}}, \end{array}$$

both Users 1 and 2 will be able to obtain the sub-file they want, i.e., $W_{1,\{2,5\}}$ and $W_{2,\{1,5\}}$, respectively. Similarly, we may consider all four users in R and the sub-files that each of them need. We find that if User {5} transmits

$$\begin{array}{c} W_{1,\{2,5\}}⊕W_{2,\{1,5\}},W_{1,\{3,5\}}⊕W_{1,\{1,5\}},\\ W_{1,\{4,5\}}⊕W_{1,\{1,5\}},W_{2,\{3,5\}}⊕W_{1,\{2,5\}},\\ W_{2,\{4,5\}}⊕W_{1,\{2,5\}},W_{1,\{4,5\}}⊕W_{1,\{3,5\}}, \end{array}$$

and User {6} transmits

$$\begin{array}{c} W_{1,\{2,6\}}⊕W_{2,\{1,6\}},W_{1,\{3,6\}}⊕W_{1,\{1,6\}},\\ W_{1,\{4,6\}}⊕W_{1,\{1,6\}},W_{2,\{3,6\}}⊕W_{1,\{2,6\}},\\ W_{2,\{4,6\}}⊕W_{1,\{2,6\}},W_{1,\{4,6\}}⊕W_{1,\{3,6\}}, \end{array}$$

all the requesting users will be able to decode the necessary sub-files of the second category. However, recall that $U^{\prime 5}=\left\{1,2\right\}$ and $U^{\prime 6}=\left\{2,3\right\}$. Hence, the signal $W_{1,\{4,5\}}⊕W_{1,\{3,5\}}$ corresponds to $\bar{B}=\left\{3,4,5\right\}$, which has zero intersection with $U^{\prime 5}$. Hence, $W_{1,\{4,5\}}⊕W_{1,\{3,5\}}$ need not be transmitted and can be calculated due to the fact that

$$\begin{array}{c} \left(W_{1,\{3,5\}}⊕W_{1,\{1,5\}}\right)⊕\left(W_{1,\{4,5\}}⊕W_{1,\{1,5\}}\right)⊕\left(W_{1,\{4,5\}}⊕W_{1,\{3,5\}}\right)=0. \end{array}$$

Similarly, $W_{1,\{4,6\}}⊕W_{1,\{1,6\}}$ need not be transmitted due to the fact that

$$\begin{array}{c} \left(W_{1,\{3,6\}}⊕W_{1,\{1,6\}}\right)⊕\left(W_{1,\{4,6\}}⊕W_{1,\{3,6\}}\right)⊕\left(W_{1,\{4,6\}}⊕W_{1,\{1,6\}}\right)=0. \end{array}$$

Hence, the rate $R^{2\mathrm{nd}}=1/15\times 5\times 2=2/3$, which coincides with (18).

For the sub-files in the third category, User 1 has R∖{1}={2,3,4}, which means $N_{e}\left(D_{R\setminus \{1\}}\right)=2$. Suppose User 1 picks the leading demanders as $U^{1}=\left\{2,4\right\}$. Similarly, User 2 has R∖{2}={1,3,4}, which means $N_{e}\left(D_{R\setminus \{1\}}\right)=1$. Suppose User 2 picks the leading demanders as $U^{2}=\left\{3\right\}$. User 3 has R∖{3}={1,2,4}, which means $N_{e}\left(D_{R\setminus \{1\}}\right)=2$. Suppose User 3 picks the leading demanders as $U^{3}=\left\{1,2\right\}$, and User 4 has R∖{4}={1,2,3}, which means $N_{e}\left(D_{R\setminus \{1\}}\right)=2$. Suppose User 4 picks the leading demanders as $U^{1}=\left\{2,3\right\}$.

Since t=2, we split the sub-files in the third category into two equal-length sub-pieces. For User 1, $E^{1}$ is of size t=2 and is a subset of R∖{1}={2,3,4}. Hence, possible $E^{1}$s can be {2,3},{2,4},and{3,4}, which all satisfy the condition of non-zero intersection with $U^{1}=\left\{2,4\right\}$. Hence, from (22) and (23), User 1 transmits

$$\begin{array}{c} Y_{\left\{2,3\right\}}^{1}=W_{2,\{1,3\},1}⊕W_{1,\{1,2\},1},Y_{\left\{2,4\right\}}^{1}=W_{2,\{1,4\},1}⊕W_{1,\{1,2\},1},Y_{\left\{3,4\right\}}^{1}=W_{1,\{1,4\},1}⊕W_{1,\{1,3\},1}. \end{array}$$

Similarly, User 3 transmits

$$\begin{array}{c} Y_{\left\{1,2\right\}}^{3}=W_{1,\{2,3\},3}⊕W_{2,\{1,3\},3},Y_{\left\{1,4\right\}}^{3}=W_{1,\{3,4\},3}⊕W_{1,\{1,3\},3},Y_{\left\{2,4\right\}}^{3}=W_{2,\{3,4\},3}⊕W_{1,\{2,3\},3}, \end{array}$$

and User 4 transmits

$$\begin{array}{c} Y_{\left\{1,2\right\}}^{4}=W_{1,\{2,4\},4}⊕W_{2,\{1,4\},4},Y_{\left\{1,3\right\}}^{4}=W_{1,\{3,4\},4}⊕W_{1,\{1,4\},4},Y_{\left\{2,3\right\}}^{4}=W_{2,\{3,4\},4}⊕W_{1,\{2,4\},4}. \end{array}$$

As for User 2, $E^{2}$ is of size t=2 and is a subset of R∖{2}={1,3,4}. Hence, possible $E^{2}$s can be {1,3},{1,4},and{3,4}. Recall that $U^{2}=\left\{3\right\}$; hence, only {1,3} and {3,4} satisfy the condition of non-zero intersection with $U^{2}=\left\{3\right\}$. Hence, from (22) and (23), User 2 transmits

$$\begin{array}{c} Y_{\left\{1,3\right\}}^{2}=W_{1,\{2,3\},2}⊕W_{1,\{1,2\},2},\;Y_{\left\{3,4\right\}}^{2}=W_{1,\{2,4\},2}⊕W_{1,\{2,3\},2}. \end{array}$$

The signal $Y_{\left\{1,4\right\}}^{2}=W_{1,\{2,4\},2}⊕W_{1,\{1,2\},2}$ does not need to be transmitted and can be calculated since $Y_{\left\{1,3\right\}}^{2}⊕Y_{\left\{3,4\right\}}^{2}⊕Y_{\left\{1,4\right\}}^{2}=0$ (cf. (24)). Hence, the rate $R^{3\mathrm{rd}}=1/30\times \left(3\times 3+2\right)=11/30$, which coincides with (25).

Thus, the total delivery rate achieved by the proposed scheme for the case R={1,2,3,4} and the request vector is $D_{\left\{1,2,3,4\right\}}=\left(1,2,1,1\right)$ is $\frac{2}{15}+\frac{2}{3}+\frac{11}{30}=\frac{7}{6}$. In this case, if we directly use the YWSC scheme [9] by assigning Users 5 and 6 a demand that is the same as Users 1, 3, and 4, i.e., all six users make file requests, and the demand vector is d={1,2,1,1,1,1}, then according to [9] (Equation (14)), the delivery rate is $\frac{6\times 10-(3\times 5+6)}{30}=\frac{13}{10}$. If we assign Users 5 and 6 with some other demands, the delivery rate will be even higher. Hence, we see that for the request-robust D2D coded caching problem, the proposed scheme performs better than directly applying the YWSC scheme for this example.

The reason why the proposed three-category-based scheme has better performance is that directly applying the YWSC scheme may contain information that is useful for users who do not request files, which is useless for requesters and difficult to be excluded. We provide more details in the following remarks:

**Remark** **6.** *When each user requests a single file (r=K), our proposed scheme corresponds to the one originally presented in [9]. The improvement in our scheme is that when r<K, we take full advantage of the users who do not request files so that the broadcast codewords are only useful to the requesters. Moreover, through the numerical comparison in Section 5, we find that letting the users who do not request files broadcast pieces of sub-files in the first and second categories, i.e., sub-files in $\left\{W_{n,A}|A∋\left\{k\right\},k\in K\setminus R\right\}$, incurs a much smaller rate than letting all the users participate in broadcasting the required pieces of sub-files in all three categories.*

**Remark** **7.** *The proposed scheme is symmetric in the placement phase. As mentioned in [9] (Remark 6), under the constraints of uncoded cache placement, the shared link models in [11,27] showed the optimality of symmetry in the placement phase [3]. This symmetry happens in the placement phase before the requesters are identified and reveal their demands, and any asymmetry in the placement will certainly not result in a better worst-case rate. However, due to the file-categorization step (i.e., Step (b)), the delivery phase of the proposed scheme is* asymmetric*, while if the value of $N_{e}\left(D_{R\setminus \{i\}}\right)$ is the same for every i∈R, the delivery phase of directly applying the YWSC scheme (i.e., the adapted YWSC scheme (see Appendix A for the specific scheme)) is symmetric. Interestingly, the asymmetric delivery phase of the proposed scheme outperforms the possibly symmetric delivery phase of directly applying the YWSC scheme both for the maximum average and worst-case delivery rate in all cases cited, as shown in Section 5.*

**Remark** **8.** *The delivery phase of the proposed scheme is actually one-shot, which is defined in [9] as meaning that each user k can recover the i-th needed bit denoted as $W_{d_{k}}^{k}\left(i\right)$ from its own cache and the transmission of a single other user whose index is $j_{k}\left(i\right)$, i.e., $H(W_{d_{k}}^{k}\left(i\right)|X_{j_{k}\left(i\right)},Z_{k})=0$ holds. One-shot delivery allows all users to participate in the transmission without causing users to repeatedly broadcast the same codeword. However, it is difficult to confirm whether the proposed scheme is optimal under the constraint of uncoded cache placement and one-shot delivery since the delivery scheme as mentioned in Remark 7 is asymmetric.*

**Remark** **9.** *The rate achieved by the three-category-based scheme from (14) outperforms the rate achieved by the adapted YWSC scheme from (A1) in some specific cases. For example, for r=2 and the maximum worst-case delivery rate, when t∈[2,K−1],r≤N, we have*

(27) Radapted-YWSCDR|r=2,t∈[2,K−1],r≤N=KK−1t−(K−2)K−2−1t−2K−2ttKt=(2K−t−1)K−2t−1tKt>(2K−t−2)K−2t−1tKt=K−2t−121+1Kt+2K−2tKt

(28) $$\begin{array}{cc} \; & =R_{\mathrm{req}-\mathrm{rob}}^{D_{R}}{|}_{r=2,t\in [2,K-1],r\leq N}, \end{array}$$

*where (27) is from (8) and (28) is from (11). Meanwhile, when t∈[2,K−1],r>N, i.e., N=1, we have*

(29) Radapted-YWSCDR|r=2,t∈[2,K−1],N=1=KK−1t−K−2ttKt=1Kt·K(K−2)!t!(K−t−1)!>1Kt·(K−1)!t!(K−t−1)!=K−2t−121+1Kt+K−2tKt

(30) $$\begin{array}{cc} \; & =R_{\mathrm{req}-\mathrm{rob}}^{D_{R}}{|}_{r=2,t\in [2,K-1],N=1}, \end{array}$$

*where (29) is from (8) and (30) is from (11). Moreover, t≥K, i.e., M≥N, is trivial, and when t=1, the three-category-based scheme and the adapted YWSC have the same performance. Hence, for r=2 and the maximum worst-case delivery rate, the three-category-based scheme outperforms the adapted YWSC scheme for all values of K,N,t.*

## 5. Numerical Evaluations

In this section, we compare the rate–memory tradeoff of the three-category-based scheme, the adapted YWSC scheme, and the achievable schemes in [8], adapted to the request-robust D2D coded caching scenario. The adaptation is performed by assigning the users, who do not request, a demand that is most requested by the requesters. We also plot the converse bounds on the optimal average and worst-case delivery rate of the request-robust D2D coded caching problem in Theorem 3.

We consider the cases where the value of *K* is from 1 to 60. For a fixed *K*, we consider that the value of *N* is from 1 to *K*. The performance metrics are the maximum average delivery rate with respect to the uniform demand and the maximum worst-case delivery rate, i.e., $R_{\mathrm{ave},\mathrm{req}-\mathrm{rob}}^{r}$ and $R_{\mathrm{worst},\mathrm{req}-\mathrm{rob}}^{r}$. We find that, in these cases, our proposed three-category-based scheme outperforms the adapted YWSC scheme and the adapted schemes of [8] for all possible *r*.

**Figure 2:**
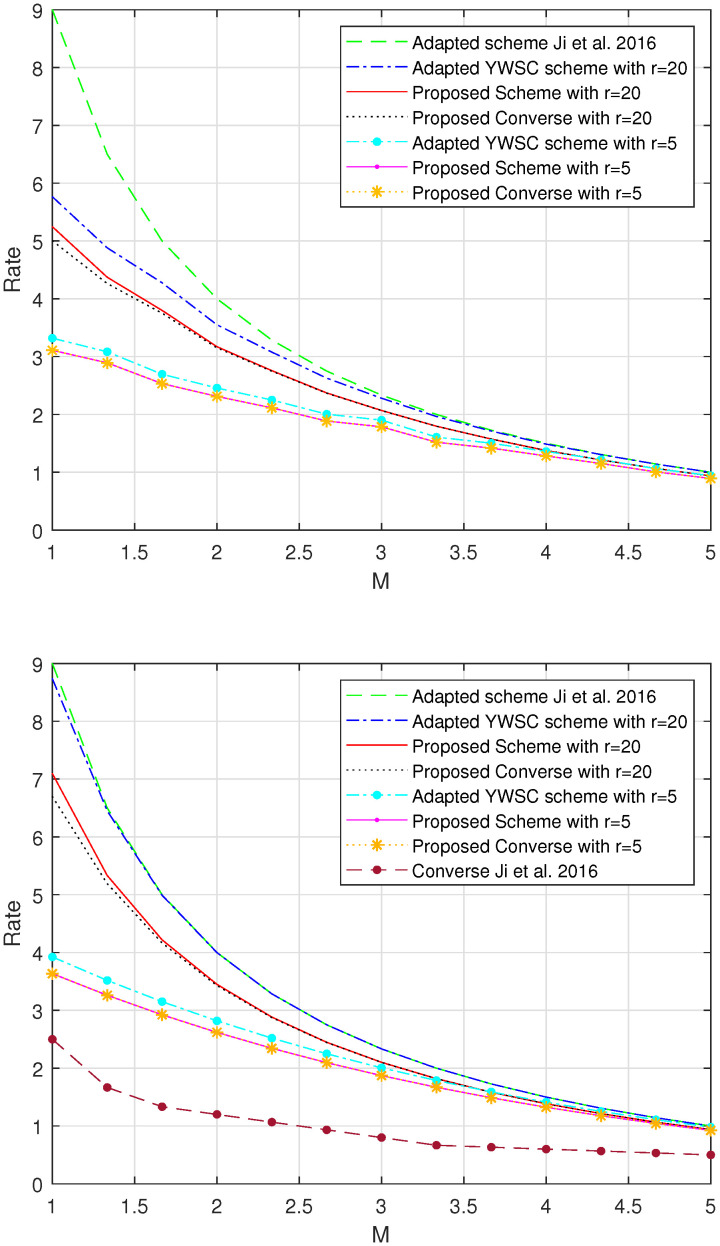
Consider the request-robust D2D coded caching problem from Section 2.1 where N=10andK=30. The figure above is for the tradeoff between memory size and the maximum worst-case delivery rate for different requester numbers. The figure below shows the tradeoff between memory size and the maximum average delivery rate under uniform demand for different requester numbers. The scheme and converse proposed by Ji et al. [8] are both adapted to this request-robust D2D scenario.

Take the example where N=10,K=30. As shown in Figure 2, the performance of the proposed scheme is given by the red solid line when r=20 and the purple solid line with dots when r=5. These lines are plotted according to the right-hand side (RHS) of (9) and (10). The performance of the adapted YWSC scheme is given by a blue dash–dot line when r=20 and a cyan dash–dot line with dots when r=5. These lines are plotted according to the RHS of (6) and (7). The proposed converse is given by the black dotted line when r=20 and the orange dotted line with asterisks when r=5. Since the achievable rate in [8] is independent of the demand, the performance of the adapted scheme from [8] does not change with the value of *r* and is given by the green dashed line. For the maximum worst-case rate, we also provide the lower bound in [8] adapted to the request-robust D2D coded caching scenario with the brown dashed line with dots. It can be seen that our proposed scheme outperforms the adapted YWSC scheme and adapted scheme of [8] in this case; meanwhile, the proposed converse is rather tight compared to the adapted lower bound in [8].

## 6. Conclusions

In this paper, we propose a new problem called request-robust D2D coded caching, where in the delivery phase, though all users in the placement phase are still present, some of them do not request any files. We presented an achievable scheme for this problem based on uncoded cache placement and exploiting common demands and one-shot delivery. The caching strategy is the same as that proposed by Maddah-Ali and Niesen, while the delivery strategy divides the sub-files into three categories, and different delivery signals are designed for each category. We also characterized information-theoretic lower bounds for the request-robust D2D coded caching problem under the constraint of uncoded cache placement. The lower bounds are both for the maximum average delivery rate under uniform demand and the maximum worst-case delivery rate. We adapt the scheme proposed by Yapar et al. for uncoded cache placement and one-shot delivery to the request-robust D2D coded caching problem. The adaptation is performed by assigning the users, who do not request, a demand that is the most requested by the requesters. The performance of the adapted scheme is proved to be order optimal within a factor of two under uncoded cache placement and within a factor of four in general. Finally, by numerical evaluation, we show that the proposed scheme outperforms the known D2D coded caching schemes applied to the request-robust scenario.

## Data Availability

Data are contained within the article.

## Appendix A. Adapted Yapar–Wan–Schaefer–Caire Scheme, i.e., Proof of Theorem 1

In this section, we present another achievable scheme that adapts the YWSC scheme from [9] for the request-robust D2D coded caching problem. The scheme achieves the rate stated in Theorem 1.

We will first provide the general achievable scheme, which is based on the uncoded cache placement and exploiting one-shot delivery [9]. Then, we will characterize the performance of the proposed scheme and show that for any requester set R and corresponding request vector $D_{R}$, the adapted YWSC scheme achieves the rate

(A1) Radapted-YWSCDR=KK−1t−∑i=1KK−1−Ne(DK∖{i}′)t−fK−rt−K−r−1ttKt,

where $D_{K}^{\prime }$ is the request vector of all the users after adaption, and *f* is an integer equal to one if and only if each requester demands a distinct file; otherwise, f=0. The rate $R_{\mathrm{adapted}-\mathrm{YWSC}}^{D_{R}}$ with the explicit characterization of the maximum worst-case delivery rate in Appendix A.2 immediately proves Theorem 1. Finally, we will provide an example to aid in a better understanding of the adapted YWSC scheme.

### Appendix A.1. General Scheme

Similar to Section 4.1, for the placement phase, we still use $M_{\mathrm{MAN}}$ described in Definition 1. In the following, we restrict to integer values of t∈[1:K]. For cache size *M* where t=KM/N is not an integer, memory-sharing will be performed [3,8].

For the delivery phase, let the set of requesters be R with size *r*. The *r* requesters each demand a single file. We find that the YWSC scheme from [9] is highly adaptable for the request-robust D2D coded caching scenario. The adaptation is performed by assigning the users, who do not request, a demand that is the most requested by the requesters. If there are multiple demands that are the most requested, the users, who do not request, would be assigned one of the demands. Recall that we denote the adapted request vector as $D_{K}^{\prime }$, and the adapted request vector of users K∖{k} as $D_{K\setminus \{k\}}^{\prime }$. For example, if $K=N=4,R=\left\{1,2\right\},D_{\left\{1,2\right\}}=\left\{1,4\right\}$, we have $D_{\left\{1,2,3,4\right\}}^{\prime }=\left\{1,4,1,1\right\}$ or $D_{\left\{1,2,3,4\right\}}^{\prime }=\left\{1,4,4,4\right\}$. Obviously, $N_{e}\left(D_{K}^{\prime }\right)=N_{e}\left(D_{R}\right)$ and $N_{e}\left(D_{K\setminus \{k\}}^{\prime }\right)\geq N_{e}\left(D_{R\setminus \{k\}}\right)$ for each k∈R.

Next, to adapt the YWSC scheme for the system model, the delivery strategy is divided into the following steps:

(a) **Determining the leading demanders**: Recall that each sub-file is denoted as $W_{n,𝒯}$ and is cached by only users in 𝒯. Each sub-file is divided into *t* equal-length disjoint sub-pieces of FtKt bits, which are denoted by $W_{n,𝒯,i},i\in 𝒯$. Further, each user *i* selects an arbitrary subset of $N_{e}\left(D_{K\setminus \{i\}}^{\prime }\right)$ users from K∖{i}, denoted by $U^{i}=\left\{u_{1}^{i},\ldots ,u_{N_{e}\left(D_{K\setminus \{i\}}^{\prime }\right)}^{i}\right\}$, which request $N_{e}\left(D_{K\setminus \{i\}}^{\prime }\right))$ distinct files and are referred to as *leading demanders of user i*.

Without a loss of generality, we assume that the leading demanders of each user *i* are determined from the requesters as much as possible and are denoted by $\bar{U^{i}}$. For example, for $K=N=3,R=\left\{1,2\right\},D_{\left\{1,2\right\}}=\left\{1,3\right\},D_{\left\{1,2,3\right\}}^{\prime }=\left\{1,3,1\right\}$, the leading demander of User 2 in the assumption is $\bar{U^{2}}=\left\{1\right\}$ and should not be {3}, while the leading demander of User 1 is $\bar{U^{1}}=\left\{2,3\right\}$.

(b) **Pre-transmitting signals**: Then, for all subset $E^{i}\subseteq K\setminus \left\{i\right\}$ of *t* users, each user *i* prepares for transmitting:
  (A2) $$\begin{array}{cc} \; & X_{i}^{\mathrm{YWSC}}={\left\{Y_{E^{i}}^{i}\right\}}_{E^{i}\cap U^{i}\neq \emptyset }, \end{array}$$
  where
  (A3) $$\begin{array}{cc} \; & Y_{E^{i}}^{i}=\underset{k\in E^{i}}{⨁}W_{d_{k},\left\{E^{i}\cup \left\{i\right\}\right\}\setminus \left\{k\right\},i}, \end{array}$$
  which is the same as (23). In other words, since all users $k\in E^{i}$ shall retrieve the needed sub-pieces $W_{d_{k},\left\{E^{i}\cup \left\{i\right\}\right\}\setminus \left\{k\right\},i}$ from the transmissions of User *i*, using the idea of leaders from [11], each user *i* only need to transmit in sequence the codewords $Y_{E^{i}}^{i}$ for all subsets $E^{i}$ such that $E^{i}\cap U^{i}\neq \emptyset$, i.e., $X_{i}^{\mathrm{YWSC}}$.
(c) **Removing unwanted codewords**: We notice that the codewords that are only useful for the users, who do not request, are unwanted codewords and do not need to be transmitted. Next, we will remove the unwanted codewords.

Recall that each user *i* only prepares for transmitting the codewords $Y_{E^{i}}^{i}$ where $E^{i}\cap \bar{U^{i}}\neq \emptyset$. For user *i*, when $\bar{U^{i}}\cap K\setminus R=\emptyset$, the signal $X_{i}^{\mathrm{YWSC}}$ does not have unwanted codewords. When $\bar{U^{i}}\cap K\setminus R\neq \emptyset$, the signal $X_{i}^{\mathrm{YWSC}}$ may have the codewords $Y_{E^{i}}^{i}$ where $E^{i}\subseteq K\setminus R$, which are only useful for the users, who do not request, and should be removed. Obviously, if and only if each requester demands a distinct file, we have $\bar{U^{i}}\cap K\setminus R\neq \emptyset$ for only one requester *i* in R. Hence, for all subsets $E^{i}\subseteq K\setminus \left\{i\right\}$ of *t* users, each user *i* transmits

$$\begin{array}{cc} \; & X_{i}^{\mathrm{adapted}-\mathrm{YWSC}}={\left\{Y_{E^{i}}^{i}\right\}}_{E^{i}\cap \bar{U^{i}}\neq \emptyset ,E^{i}⊈K\setminus R}, \end{array}$$

where $Y_{E^{i}}^{i}$ is defined in (A3).

As a result, when Requester *k* is a leading demander for User *i*, i.e., $k\in U^{i}$, it can decode any needed sub-piece $W_{d_{k},G^{k}\cup \left\{i\right\},i}$, where $G^{k}\subseteq K\setminus \left\{i,k\right\}$, $|G^{k}|=t-1$, from $Y_{G^{k}\cup \left\{k\right\}}^{i}$, which is transmitted from User *i*, by performing

$$W_{d_{k},G^{k}\cup \left\{i\right\},i}=\left(\underset{x\in G^{k}}{⨁}W_{d_{x},\left\{G^{k}\cup \left\{i,k\right\}\right\}\setminus \left\{x\right\},i}\right)⨁Y_{G^{k}\cup \left\{k\right\}}^{i}.$$

When Requester *k* is not a leading demander for User *i*, using the equation

$$\underset{V^{i}\in V_{F}^{i}}{⨁}Y_{C^{i}\setminus V^{i}}^{i}=0,$$

proved in [9] (Lemma 1), where subset $C^{i}\subseteq K\setminus \left\{i\right\}$ includes $\bar{U^{i}}$ and $V_{F}^{i}$ denotes the family of all subsets $V^{i}$ of $C^{i}$ such that each requested file in $D_{K\setminus \{i\}}^{\prime }$ is requested by exactly one user in $V^{i}$, each user *k* can decode its requested sub-piece through obtaining the value $Y_{E^{i}}^{i}$ for any subset $E^{i}$ of *t* users such that $E^{i}\cap \bar{U^{i}}=\emptyset$ from the broadcast codewords by the following equation:

$$Y_{E^{i}}^{i}=\underset{V^{i}\in V_{F}^{i}\setminus \left\{\bar{U^{i}}\right\}}{⨁}Y_{C^{i}\setminus V^{i}}^{i},$$

where $C^{i}=E^{i}\cup \bar{U^{i}}$. To sum up, for each i∈K∖{k}, Requester *k* decodes its requested sub-pieces by following either one of the strategies above depending on whether it is a leading demander of User *i* or not.

For each user i∈K, the size of the transmitted signal amounts to K−1t−K−1−Ne(DK∖{i}′)t times the size of a sub-piece. Meanwhile, if we have $\bar{U^{i}}\cap K\setminus R\neq \emptyset$ for only one requester *i* in R, the size of the removed unwanted codewords amounts to K−rt−K−r−1t times the size of a sub-piece. Hence, the rate of transmitting total bits coincides (A1).

For the convenience of understanding, we give the algorithm of the adapted YWSC scheme in Algorithm A1.

**Algorithm A1** Adapted YWSC Scheme (N,K,M)
procedure PLACEMENT($W_{1},\ldots ,W_{N}$) 1: Apply Algorithm 1 MAN Uncoded Symmetric Placement Scheme($N,K,M,W_{\left[N\right]}$) procedure DELIVERY($R,D_{R}$) 2: $r\leftarrow |D_{R}|$ 3: t←KM/N 4: Assign the users, who do not request, a demand that is the most requested by the requesters and has $D_{K}^{\prime }\leftarrow D_{R}$ 5: 𝒢 ←{G⊆[K]:|G|=t} 6: **for** n∈[N] **do** 7: **for** G∈ 𝒢 **do** 8: Divide sub-file $W_{n,G}$ into *t* disjoint sub-pieces $\left(W_{n,G,i}:i\in G\right)$ with equal size 9: **end for** 10: **end for** 11: **for** i∈K **do** 12: $N_{e}\left(D_{K\setminus \{i\}}^{\prime }\right)\leftarrow$ the number of distinct elements in $D_{K\setminus \{i\}}^{\prime }$ 13: $\bar{U^{i}}\leftarrow \left\{u_{1}^{i},...,u_{N_{e}\left(D_{K\setminus \{i\}}^{\prime }\right)}^{i}\right\}$ from R as much as possible 14: **for** $E^{i}\subseteq K\setminus \left\{i\right\}:\left|E^{i}\right|=t$ users **do** 15: **if** $E^{i}\cap \bar{U^{i}}==\emptyset$ **then** 16: continue 17: **else if** $E^{i}\subseteq K\setminus R$ **then** 18: continue 19: **else** 20: User *i* transmits $Y_{E^{i}}^{i}=\underset{k\in E^{i}}{⨁}W_{d_{k},\left\{E^{i}\cup \left\{i\right\}\right\}\setminus \left\{k\right\},i}$ 21: **end if** 22: **end for** 23: **end for**

### Appendix A.2. The Maximum Worst-Case Delivery Rate

In this section, we characterize the performance of the adapted YWSC scheme for the maximum worst-case delivery rate. The characterization is based on the observation that the binomial coefficient nm demonstrates a strictly ascending pattern with respect to *n*.

Again, the adaptation is performed by assigning the users, who do not request, a demand that is the most requested by the requesters, and we have $N_{e}\left(D_{K}^{\prime }\right)=N_{e}\left(D_{R}\right)$. Meanwhile, *f* from (A1) is an integer equal to one if and only if each requester demands a distinct file, i.e., $N_{e}\left(D_{R}\right)=r$; otherwise, f=0. Hence, for the request-robust D2D coded caching problem, when N,K,M,r,andR do not change, since the upper bound rate $R_{\mathrm{adapted}-\mathrm{YWSC}}^{D_{R}}$ from (A1) decreases as $N_{e}\left(D_{K}^{\prime }\right)$ decreases, the upper bound rate for the maximum worst-case delivery rate is the one at the maximum value of $N_{e}\left(D_{K}^{\prime }\right)$, i.e., $N_{e}\left(D_{K}^{\prime }\right)=\min\left\{r,N\right\}.$

Then, for r≥2N, each file can be requested by at least two users, which leads to $N_{e}\left(D_{K\setminus \{k\}}^{\prime }\right),\forall k\in K$ having the maximum value of *N*. Meanwhile, in this case, f=0. Hence, this case maximizes the upper bound rate $R_{\mathrm{adapted}-\mathrm{YWSC}}^{D_{R}}$.

For r<2N, User *k* may be the only user requesting a file or not, which leads to $N_{e}\left(D_{K\setminus \{k\}}^{\prime }\right)=N_{e}\left(D_{K}^{\prime }\right)-1$ and $N_{e}\left(D_{K\setminus \{k\}}^{\prime }\right)=N_{e}\left(D_{K}^{\prime }\right)$, respectively. Due to the adaption, for r≤N, there are r−1 users, each of which are the only users requesting a file while each of the remaining K−r+1 users are not. Meanwhile, in the case r≤N, $N_{e}\left(D_{K}^{\prime }\right)=r$ and f=1. Thus, we prove the case where r≤N.

For N<r<2N, we have $N_{e}\left(D_{K}^{\prime }\right)=N$. Then, for $D_{R}$, we obtain that each file cannot be requested by more than two requesters. Thus, due to a total of *K* users, there are 2N−r users, each of which are the only users requesting a file while each of the remaining K+r−2N users are not. Meanwhile, in this case, f=0. Thus, we prove the case where N<r<2N.

### Appendix A.3. Example

To aid in better understanding, we provide an example to illustrate the adapted YWSC scheme in Appendix A.1.

Let us consider a case where N=2,K=4,andM=1. Hence, t=KM/N=2. In the placement phase, each file is divided into 42=6 sub-files, and each sub-file’s index is in T, where T is the family of all sets 𝒯 such that 𝒯⊂[4],|𝒯|=2. The user k∈[6] caches the following sub-files for each n∈{1,2}:

$$Z_{k}=\left\{W_{n,𝒯}|𝒯\in T,k\in 𝒯\right\}.$$

In the delivery phase, without a loss of generality, we only consider Users 1and2, as requesters, each requesting a single file, i.e., R={1,2}, and the request vector is $D_{\left\{1,2\right\}}=\left(1,2\right)$. Hence, we consider that the request vector after adaption is $D_{\left\{1,2,3,4\right\}}^{\prime }=\left(1,2,1,1\right)$. Notice that r=4 and $N_{e}\left(D_{\left\{1,2,3,4\right\}}^{\prime }\right)=2$. Requesters 1and2 need the following missing sub-files:

$$\begin{array}{c} W_{1}\setminus Z_{1}=\left\{W_{1,\{2,3\}},W_{1,\{2,4\}},W_{1,\{3,4\}}\right\},\\ W_{2}\setminus Z_{2}=\left\{W_{2,\{1,3\}},W_{2,\{1,4\}},W_{2,\{3,4\}}\right\}. \end{array}$$

In Step (a), since t=2, we split the sub-files into two equal-length sub-pieces. Further, determining the leading demanders from the requesters as much as possible, without a loss of generality, we assume that User 1 picks Users 1and3, User 2 picks User 1, User 3 picks Users 1and2, and User 4 picks Users 1and2 as the leading demanders, i.e., $\bar{U^{1}}=\left\{2,3\right\},\bar{U^{2}}=\left\{1\right\},\bar{U^{3}}=\left\{1,2\right\},and\;\bar{U^{4}}=\left\{1,2\right\}$. Notice that $N_{e}\left(D_{K\setminus \{1\}}^{\prime }\right)=2,N_{e}\left(D_{K\setminus \{2\}}^{\prime }\right)=1,N_{e}\left(D_{K\setminus \{3\}}^{\prime }\right)=2,and\;N_{e}\left(D_{K\setminus \{4\}}^{\prime }\right)=2$.

In Step (b), for User 1, $E^{1}$ is of size t=2 and is a subset of K∖{1}={2,3,4}. Hence, possible $E^{1}$s can be {2,3},{2,4},and{3,4}, which all satisfy the condition of non-zero intersection with $\bar{U^{1}}=\left\{2,3\right\}$. Hence, from (22) and (23), User 1 pre-transmits

$$\begin{array}{c} Y_{\left\{2,3\right\}}^{1}=W_{2,\{1,3\},1}⊕W_{1,\{1,2\},1},Y_{\left\{2,4\right\}}^{1}=W_{2,\{1,4\},1}⊕W_{1,\{1,2\},1},Y_{\left\{3,4\right\}}^{1}=W_{1,\{1,4\},1}⊕W_{1,\{1,3\},1}. \end{array}$$

Similarly, User 3 pre-transmits

$$\begin{array}{c} Y_{\left\{1,2\right\}}^{3}=W_{1,\{2,3\},3}⊕W_{2,\{1,3\},3},Y_{\left\{1,4\right\}}^{3}=W_{1,\{3,4\},3}⊕W_{1,\{1,3\},3},Y_{\left\{2,4\right\}}^{3}=W_{2,\{3,4\},3}⊕W_{1,\{2,3\},3}, \end{array}$$

and User 4 pre-transmits

$$\begin{array}{c} Y_{\left\{1,2\right\}}^{4}=W_{1,\{2,4\},4}⊕W_{2,\{1,4\},4},Y_{\left\{1,3\right\}}^{4}=W_{1,\{3,4\},4}⊕W_{1,\{1,4\},4},Y_{\left\{2,3\right\}}^{4}=W_{2,\{3,4\},4}⊕W_{1,\{2,4\},4}. \end{array}$$

As for User 2, $E^{2}$ is of size t=2 and is a subset of K∖{2}={1,3,4}. Hence, possible $E^{2}$s can be {1,3},{1,4},and{3,4}. Recall that $\bar{U^{2}}=\left\{1\right\}$; hence, only {1,3} and {1,4} satisfy the condition of non-zero intersection with $U^{2}=\left\{1\right\}$. Hence, from (A2) and (A3), User 2 pre-transmits

$$\begin{array}{c} Y_{\left\{1,3\right\}}^{2}=W_{1,\{2,3\},2}⊕W_{1,\{1,2\},2},\;Y_{\left\{1,4\right\}}^{2}=W_{1,\{2,4\},2}⊕W_{1,\{1,2\},2}. \end{array}$$

The signal $Y_{\left\{3,4\right\}}^{2}=W_{1,\{2,3\},2}⊕W_{1,\{2,4\},2}$ does not need to be pre-transmitted and can be calculated since $Y_{\left\{1,3\right\}}^{2}⊕Y_{\left\{1,4\right\}}^{2}⊕Y_{\left\{3,4\right\}}^{2}=0$ (cf. (24)).

In Step (c), removing unwanted codewords, we notice that the codeword $Y_{\left\{3,4\right\}}^{1}$ is only useful for the users, who do not request, and hence are removed. The other codewords are transmitted in the way of Step (b).

Thus, the total delivery rate achieved by the adapted YWSC scheme for the case R={1,2} and $D_{\left\{1,2,3,4\right\}}^{\prime }=\left(1,2,1,1\right)$ is $R_{\mathrm{adapted}-\mathrm{YWSC}}^{\left\{1,2\right\}}=\frac{2+3+3+2}{12}=\frac{5}{6}$, which coincides with (A1). In this case, if we directly use the YWSC scheme [9] by assigning Users 3 and 4 a demand that is the same as User 1, then according to [9] (Equation (14)), the delivery rate is $\frac{4\times 3-1}{12}=\frac{11}{12}$. If we assign Users 3 and 4 with some other demands, the delivery rate will be the same. However, if we use the proposed three-category-based scheme from Section 4.1, then according to (A1), the delivery rate is $R_{\mathrm{req}-\mathrm{rob}}^{\left\{1,2\right\}}=\frac{2}{3}$. Hence, we see that for the request-robust D2D coded caching problem, the proposed three-category-based scheme performs better than the adapted YWSC scheme for this example.

The reason why the proposed three-category-based scheme and the adapted YWSC scheme have better performance is in the following remark:

**Remark** **A1.**
*The achievable rate $R_{\mathrm{adapted}-\mathrm{YWSC}}^{D_{R}}$ of our model is similar to the achievable rate $R^{*}\left(d,M_{\mathrm{MAN}}\right)$ of Yapar’s model [9]. The main difference is that the value of $N_{e}\left(d_{\setminus \{i\}}\right)$ in $R^{*}\left(d,M_{\mathrm{MAN}}\right)$ depends on all the users’ request vector d, while the value of $N_{e}\left(D_{K\setminus \{i\}}^{\prime }\right)$ in $R_{\mathrm{adapted}-\mathrm{YWSC}}^{D_{R}}$ depends on the r requesters’ request vector $D_{R}$. When N,K,andM is the same, for User i, there is $N_{e}\left(D_{K\setminus \{i\}}^{\prime }\right)\leq N_{e}\left(d_{\setminus \{i\}}\right)$, and hence $R_{\mathrm{adapted}-\mathrm{YWSC}}^{D_{R}}\leq R^{*}\left(d,M_{\mathrm{MAN}}\right)$ for uniform demand distribution and the worst case. However, using the adapted YWSC scheme, the signal $X_{i}^{\mathrm{adapted}-\mathrm{YWSC}}$ may contain useless information. For example, in Appendix A.3, the signal $Y_{\left\{2,3\right\}}^{1}$ contains the useless sub-piece $W_{1,\{1,2\},1}$, which is transmitted for User 3, who does not request. We find that removing useless sub-pieces does not affect the size of the rate but makes signals contain too small uncoded sub-pieces. The proposed three-category-based scheme in Section 4 does not contain useless information and maximizes the global gain brought from the code by utilizing the users, who do not request, to transmit signals as much as possible.*

## Appendix B. Proof of Theorem 3

### Appendix B.1. The Maximum Average Delivery Rate

In this subsection, we propose the converse bound given in Theorem 3 for the maximum average delivery rate of the request-robust D2D coded caching problem. Let us consider the problem of coded caching with inactive users defined in Section 2.2.2 first. For a problem of coded caching with inactive users with the rate $R_{\mathrm{inact}}$, given the same placement *Z* and requester demand vector $D_{R}$ as in Section 2.1, the encoding function on the central server is

$$\phi^{\prime D_{R}}:\left[2^{NF}\right]\to \left[2^{R_{\mathrm{inact}}F}\right],$$

and the delivery information $X^{\prime }$ is denoted as $X^{\prime }=\phi^{\prime D_{R}}\left(W_{1},\ldots ,W_{N}\right)$. The *r* requesters decode the request message according to caching content and delivery information, and the decoding function is defined as

$$\psi_{k}^{\prime D_{R}}:\left[2^{M_{k}F}\right]\times \left[2^{R_{\mathrm{inact}}F}\right]\to \left[2^{F}\right],\;k\in R.$$

The file decoded by User *k* is denoted as ${\hat{W}}_{d_{k}}=\psi_{k}^{\prime D_{R}}\left(X^{\prime },Z_{k}\right)$. If the error probability of this system satisfies

$$P({\hat{W}}_{d_{k}}\neq W_{d_{k}})\leq \epsilon ,$$

we call the system *ϵ-achievable*.

Given $D_{R}$ and Z, we define the minimum achievable rate of the *ϵ-achievable* system as $R_{\epsilon ,\mathrm{inact}}^{D_{R}*}\left(Z\right)$. For a fixed R with size *r*, $D^{R}$ is defined as the set of all possible demands ${\left\{1,\cdots ,N\right\}}^{r}$. When $D_{R}$ is uniformly distributed on $D^{R}$, given placement *Z*, the average delivery rate with respect to the uniform demand $R_{\epsilon ,\mathrm{ave},\mathrm{inact}}^{R*}\left(Z\right)$ is defined as

$$R_{\epsilon ,\mathrm{ave},\mathrm{inact}}^{R*}\left(Z\right)=E_{D_{R}}\left[R_{\epsilon ,\mathrm{inact}}^{D_{R}*}\left(Z\right)\right].$$

For a given *r*, we define the maximum average delivery rate with respect to the uniform demand, denoted as $R_{\mathrm{ave},\mathrm{inact}}^{r}$, where the maximum is over all the request sets R with size *r*. Then, the optimal maximum average delivery rate of the memory–rate tradeoff is essentially the maximum average delivery rate for the minimum value given an arbitrary cache size *M*, and at this rate, the user can decode the requested file with a sufficiently large size without error; that is,

$$R_{\mathrm{ave},\mathrm{inact}}^{r*}=\underset{\epsilon >0}{\sup}\;\underset{F\to +\infty }{\lim\;\sup}\;\min_{Z}\max_{R:|R|=r}R_{\epsilon ,\mathrm{ave},\mathrm{inact}}^{R*}\left(Z\right).$$

Similarly, for a fixed R with size *r*, given placement *Z*, the worst-case delivery rate is

$$R_{\epsilon ,\mathrm{worst},\mathrm{inact}}^{R*}\left(Z\right)=\max_{D_{R}}R_{\epsilon ,\mathrm{inact}}^{D_{R}*}\left(Z\right),$$

and for a given *r*, the optimal maximum worst-case delivery rate we want to find is

$$R_{\mathrm{worst},\mathrm{inact}}^{r*}=\underset{\epsilon >0}{\sup}\;\underset{F\to +\infty }{\lim\;\sup}\;\min_{Z}\max_{R:|R|=r}R_{\epsilon ,\mathrm{worst},\mathrm{inact}}^{R*}\left(Z\right).$$

For the problem of coded caching with inactive users, we have the following results:

**Theorem** **A1.**
*For the problem of coded caching with inactive users with K users, a database of N files, uncoded cache sizes of M files at each user, only r users as requesters demanding files during the delivery phase, and parameter $t=\frac{KM}{N}$, we have*

(A4)
Rave,inactr*=EDRKt+1−K−Ne(DR)t+1Kt,

*for t∈K, where $D_{R}$ is uniformly random on $D^{R}={\left\{1,...,N\right\}}^{r}$ and $N_{e}\left(D_{R}\right)$ denotes the number of distinct requests in $D_{R}$. When t∉K, $R_{\mathrm{ave},\mathrm{inact}}^{r*}$ equals the lower convex envelope of the values in (A4) for integer values of t∈K.*

*Moreover, for the worst-case rate, we have*

(A5)
Rworst,inactr*=Kt+1−K−min{r,N}t+1Kt,

*for t∈K. When t∉K, $R_{\mathrm{worst},\mathrm{inact}}^{r*}$ equals the lower convex envelope of the values in (A5) for integer values of t∈K.*

**Proof.** The tight lower bounds of the average delivery rate and worst-case delivery rate are derived in the rest of this section. The caching and delivery scheme that achieves the optimal maximum average and worst-case rates is described in Appendix C. □

Due to inequality (4), to prove Theorem 3, we just need to prove Theorem A1. Motivated by [11], to lower bound the achievable average rate, we first introduce the concept of demand-type division. For the problem of coded caching with inactive users with the number of initial users *K*, the number of requesters *r*, and the fixed request set R, given the request vector $D_{R}$, we define $s_{R}\left(D_{R}\right)$ as its *statistics* such that the $i^{\mathrm{th}}$ element of $s_{R}\left(D_{R}\right)$ is equal to the number of the *i*-th most requested file. For example, when K=6andN=4, and during the delivery phase $r=4,R=\left\{1,2,3,4\right\},and\;D_{R}=\left\{1,1,3,4\right\}$, the statistic for $D_{R}$ is $s_{\left\{1,2,3,4\right\}}\left(D_{R}\right)=\left(2,1,1,0\right)$. For convenience, we simply the statistics $s_{R}\left(D_{R}\right)$ as $s_{R}$. Then, we denote the set of all possible statistics by $S_{R}$. Through this statistical method, the set of request vectors $D^{R}$ can be divided into some subsets as *type*, and type $D^{s_{R}}$ is defined as the set of queries with statistics $s_{R}$.

Note that for each request vector $D_{R}$, the value of $N_{e}\left(D_{R}\right)$ only depends on its statistic $s_{R}\left(D_{R}\right)$, so for the same type of request vector, the rate is the same. For convenience, the $N_{e}\left(D_{R}\right)$ of the type $D^{s_{R}}$ is called $N_{e}\left(s_{R}\right)$.

Given the number of requesters *r*, the request set R, and the placement *Z*, for each type $D^{s_{R}}$ in the problem of coded caching with inactive users, the average delivery rate $R_{\epsilon ,\mathrm{ave},\mathrm{inact}}^{s_{R}*}\left(Z\right)$ is defined as

$$R_{\epsilon ,\mathrm{ave},\mathrm{inact}}^{s_{R}*}\left(Z\right)=\frac{1}{|D^{s_{R}}|}\sum_{D_{R}\in D^{s_{R}}}R_{\epsilon ,\mathrm{inact}}^{D_{R}*}\left(Z\right),$$

For all types of request vectors, we have

(A6) $$\begin{array}{cc} R_{\mathrm{ave},\mathrm{inact}}^{r*} & =\underset{\epsilon >0}{\sup}\;\underset{F\to +\infty }{\lim\;\sup}\;\min_{Z}\max_{R:|R|=r}\;E_{s_{R}}\left[R_{\epsilon ,\mathrm{ave},\mathrm{inact}}^{s_{R}*}\left(Z\right)\right]\\ \; & \geq \underset{\epsilon >0}{\sup}\;\underset{F\to +\infty }{\lim\;\sup}\max_{R:|R|=r}\;E_{s_{R}}\left[\min_{Z}\;R_{\epsilon ,\mathrm{ave},\mathrm{inact}}^{s_{R}*}\left(Z\right)\right]. \end{array}$$

Hence, the lower bound of $R_{\mathrm{ave},\mathrm{inact}}^{r*}$ can be derived through bounding the minimum value of $R_{\epsilon ,\mathrm{ave},\mathrm{inact}}^{s_{R}*}\left(Z\right)$ for each type $D^{s_{R}}$ individually. We use the following lemma to lower bound the average rates within each type:

**Lemma** **A1.**
*Consider the problem of coded caching with inactive users with K initial users, N files, MF cache sizes, r requesters, and request set R during the delivery phase; for each type $D^{s_{R}}$, the minimum value of $R_{\epsilon ,\mathrm{ave},\mathrm{inact}}^{s_{R}*}\left(Z\right)$ is lower bounded by*

(A7)
minZRϵ,ave,inactsR*(Z)≥ConvKt+1−K−Ne(sR)t+1Kt−1F+Ne2(sR)ϵ,

*where Conv(f(t)) denotes the lower convex envelope of the following points: {(t,f(t))|t∈{0,1,...,K}}.*

**Proof.** We notice that solving the fundamental limits of the problem of coded caching with inactive users is essentially the caching problem defined in [11]. The only difference between the problem of coded caching with inactive users and the caching problem defined in [11] is the size of the request vector. Therefore, applying [11] (Lemma 2) and replacing the caching problem parameters demand d; statistics of demand s; set of all possible statistics S; set of all possible demands D; and type of demand $D_{s}$ with $D_{R},s_{R}\left(D_{R}\right),S_{R},D^{R},and\;D^{s_{R}}$, we obtain (A7). □

From (A6) and Lemma A1, the lower bound of $R_{\mathrm{ave},\mathrm{inact}}^{r*}$ can be further derived as

(A8) Rave,inactr*≥EsRConvKt+1−K−Ne(sR)t+1Kt.

Because the sequence

cn=Kn+1−K−Ne(sR)n+1Kn,

is convex, the order of the expectation and the Conv in (A8) can be switched. Therefore, $R_{\mathrm{ave},\mathrm{inact}}^{r*}$ is lower bounded by the rate defined in Theorem A1, which immediately proves Theorem 3 for the maximum average delivery rate.

### Appendix B.2. The Maximum Worst-Case Delivery Rate

Due to (5), to prove Theorem 3 for the maximum worst-case delivery rate, we just need to prove Theorem A1 for the maximum worst-case delivery rate. Given the number of requesters *r*, the request set R, and placement *Z*, for each type $D^{s_{R}}$ in the problem of coded caching with inactive users, we define the worst-case delivery rate $R_{\epsilon ,\mathrm{worst},\mathrm{inact}}^{s_{R}*}\left(Z\right)$ as

$$R_{\epsilon ,\mathrm{worst},\mathrm{inact}}^{s_{R}*}\left(Z\right)=\max_{D_{R}\in D^{s_{R}}}R_{\epsilon ,\mathrm{inact}}^{D_{R}*}\left(Z\right).$$

Note that

$$\begin{array}{cc} R_{\mathrm{worst},\mathrm{inact}}^{r*} & =\underset{\epsilon >0}{\sup}\;\underset{F\to +\infty }{\lim\;\sup}\;\min_{Z}\;\max_{R:|R|=r}\;\max_{s_{R}}\;R_{\epsilon ,\mathrm{worst},\mathrm{inact}}^{s_{R}*}\left(Z\right)\\ \; & \geq \underset{\epsilon >0}{\sup}\;\underset{F\to +\infty }{\lim\;\sup}\;\max_{R:|R|=r}\;\max_{s_{R}}\;\min_{Z}\;R_{\epsilon ,\mathrm{worst},\mathrm{inact}}^{s_{R}*}\left(Z\right). \end{array}$$

For each $s_{R}\in S_{R}$, using Lemma A1, we can know that

minZRϵ,worst,inactsR*(Z)≥minZRϵ,ave,inactsR*(Z)≥ConvKt+1−K−Ne(sR)t+1Kt−1F+Ne2(sR)ϵ.

When N,K,M,r,andR do not change, since the worst-case delivery rate $R_{\epsilon ,\mathrm{worst},\mathrm{inact}}^{s_{R}*}\left(Z\right)$ decreases as $N_{e}\left(s_{R}\right)$ decreases, the optimal worst-case delivery rate is the one at the maximum value of $N_{e}\left(s_{R}\right)$. Meanwhile, there is

(A9) $$\begin{array}{c} N_{e}\left(s_{R}\right)\leq \min\left\{r,N\right\}. \end{array}$$

Consequently,

Rworst,inactr*≥supϵ>0limsupF→+∞maxR:|R|=rmaxsRConvKt+1−K−Ne(sR)t+1Kt−1F+Ne2(sR)ϵ=ConvKt+1−K−min{r,N}t+1Kt.

Therefore, $R_{\mathrm{worst},\mathrm{inact}}^{r*}$ is lower bounded by the rate defined in Theorem A1 for the maximum worst-case delivery rate, which immediately proves Theorem 3 for the maximum worst-case delivery rate.

## Appendix C. Optimal Scheme for Problem of Coded Caching with Inactive Users Achieved in Theorem A1

In this section, we present the optimal scheme for the problem of coded caching with inactive users. The scheme achieves the rate stated in Theorem A1. We will characterize the performance of the proposed scheme and show that for any requester set R and corresponding request vector $D_{R}$, the optimal scheme achieves the rate

(A10) Rinact=Kt+1−K−Ne(DR)t+1Kt.

The rate $R_{\mathrm{inact}}$ with the explicit characterization of the maximum worst-case delivery rate in (A9) immediately proves Theorem A1.

Similar to Section 4.1, we restrict to integer values of t∈K and use the MAN uncoded symmetric placement scheme given in Definition 1 in the placement phase. For non-integer values of *t*, the pair $\left(M,R_{\mathrm{inact}}\right)$ achieves the lower convex envelope of the achievable points for integer values of t∈K.

For the delivery phase, the central server arbitrarily selects a subset of $N_{e}\left(D_{R}\right)$ requesters, denoted by $\hat{U}=\left\{{\hat{u}}_{1},\ldots ,{\hat{u}}_{N_{e}\left(D_{R}\right)}\right\}$, that requests $N_{e}\left(D_{R}\right)$ distinct files, $\hat{U}\subseteq R$. Following the idea of leaders from [11], we name these requesters as *leaders*.

Given an arbitrary subset H of t+1 users, each requester k∈H∩R needs the sub-file $W_{d_{k},H\setminus \left\{k\right\}}$, which is known by all other users in H. Precisely, all the requesters k∈H∩R shall retrieve the needed sub-files $W_{d_{k},H\setminus \left\{k\right\}}$ from the transmissions of the central server. By letting the central server broadcast the codeword

(A11) $$Y_{H}=\underset{x\in H\cap R}{⨁}W_{d_{x},H\setminus \left\{x\right\}},\;\mathrm{where}\;H\cap R\neq \emptyset ,$$

this sub-file retrieval can be accomplished since each requester k∈H∩R has all the sub-files on the RHS of (A11) except for $W_{d_{k},H\setminus \left\{k\right\}}$.

In order to achieve the rate in (A10), the central server only needs to transmit the codeword $Y_{H}$, which is useful for at least one leader, i.e., $X^{\mathrm{inact}}={\left\{Y_{H}\right\}}_{H\cap \hat{U}\neq \emptyset }$. The above delivery scheme totally transmits Kt+1−K−Ne(DR)t+1 codewords each with a size of F/Kt bits, which achieves the rate in (A10).

**Remark** **A2.**
*We notice that the proposed scheme is in fact the shared-link model [3] with the same file number N, file size F, user number K, cache size M, and corresponding parameter t. The only difference is the size of the request vector. Replacing the shared-link system parameter $N_{e}\left(d\right)$ in (19) with $N_{e}\left(D_{R}\right)$, we obtain (A10).*

We now prove that each requester can decode the file requested by the above delivery scheme. When *k* is a leader, i.e., $k\in \hat{U}$, it can decode any required sub-file $W_{d_{k},H^{\prime }}$, where $H^{\prime }∋k,\left|H^{\prime }\right|=t$, from $Y_{H^{\prime }\cup \left\{k\right\}}$ by performing

(A12) $$W_{d_{k},H^{\prime }}=\left(\underset{x\in H^{\prime }}{⨁}W_{d_{x},H^{\prime }\cup \left\{k\right\}\setminus \left\{x\right\}}\right)⨁Y_{H^{\prime }\cup \left\{k\right\}},$$

where $Y_{H^{\prime }\cup \left\{k\right\}}$ is defined in (A11).

When $k\in R\setminus \hat{U}$, not all codewords $Y_{H^{\prime }\cup \left\{k\right\}}$ are transmitted, and the requester *k* needs to decode the required codewords not transmitted directly. We use Lemma A2 to explain that the non-leader requester *k* can also decode all the required sub-files, even if the central server does not transmit $Y_{H^{\prime }\cup \left\{k\right\}}$, where $H^{\prime }\cap \hat{U}=\emptyset$.

**Lemma** **A2.**
*Given the request vector $D_{R}$ and picking a set of leaders $\hat{U}$, for any set I⊆K, let $V_{F}$ be the family of all subsets V of I such that each requested file in $D_{R}$ is requested by exactly one user in V, and we have*

(A13)
$$\underset{V\in V_{F}}{⨁}Y_{I\setminus V}=0,$$

*where $Y_{I\setminus V}$ is defined in (A11).*

**Proof.** As mentioned in Remark A2, the proposed scheme for the problem of coded caching with inactive users is actually the shared-link scheme. Thus, Lemma 1 in [11] can be directly applied to the proposed scheme. □

Consider any subset H of t+1 non-leader users but containing requesters. From Lemma A2, the codeword $Y_{H}$ can be directly computed from the transmitted codewords by using the following equation:

(A14) $$Y_{H}=\underset{V\in V_{F}\setminus \left\{\hat{U}\right\}}{⨁}Y_{I\setminus V},$$

where $I=H\cup \hat{U}$, given the fact that all codewords on the RHS of (A14) are broadcast, because each I∖V has a size of t+1 and contains at least one leader. Hence, each requester *k* can obtain the value $Y_{H}$ for any subset H, where H∩R≠∅ of t+1 users, and can decode its demanded files as discussed in (A11). The proposed scheme for the problem of coded caching with inactive users achieves the rate in (A10), which proves the achievability of Theorem A1.

For the convenience of understanding, we give the algorithm of the caching and delivery scheme with inactive users in Algorithm A2.

**Algorithm A2** Caching and Delivery Scheme with Inactive Users (N,K,M)
procedure PLACEMENT ($W_{1},\ldots ,W_{N}$) 1: Apply Algorithm 1 MAN Uncoded Symmetric Placement Scheme ($N,K,M,W_{\left[N\right]}$) procedure DELIVERY ($R,D_{R}$) 2: t←KM/N 3: $N_{e}\left(D_{R}\right)\leftarrow$ the number of distinct elements in $D_{R}$ 4: $\hat{U}\leftarrow \left\{{\hat{u}}_{1},...,{\hat{u}}_{N_{e}\left(D_{R}\right)}\right\}$ 5: **for** H⊆[K]:|H|=t+1 **do** 6: **if** $H\cap \hat{U}==\emptyset$ **then** 7: continue 8: **else** 9: Central server transmits $Y_{H}=\underset{x\in H:d_{x}\in D_{R}}{⨁}W_{d_{x},H\setminus \left\{x\right\}}$ 10: **end if** 11: **end for**

We provide an example to explain how the proposed scheme works.

Let us consider a case that N=2,K=3,M=2/3,andt=KM/N=1. In the placement phase, each file is divided into 31=3 sub-files. The users k∈[3] cache the following sub-files:

$$Z_{1}=\left\{W_{1,\{1\}},W_{2,\{1\}}\right\},\;Z_{2}=\left\{W_{1,\{2\}},W_{2,\{2\}}\right\},\;Z_{3}=\left\{W_{1,\{3\}},W_{2,\{3\}}\right\}.$$

In the delivery phase, only User 1and2 as requesters request a single file, i.e., R={1,2}, and the request vector is $D_{\left\{1,2\right\}}=\left(1,1\right)$. Notice that r=2 and $N_{e}\left(D_{\left\{1,2\right\}}\right)=1$.

Without a loss of generality, assume that the central server picks User 1 as the leader, i.e., $\hat{U}=\left\{1\right\}$. Then, the central server transmits the following codewords:

$$\begin{array}{c} Y_{\left\{1,2\right\}}=W_{1,\{2\}}⊕W_{1,\{1\}},\;Y_{\left\{1,3\right\}}=W_{1,\{3\}}, \end{array}$$

without transmitting $Y_{\left\{2,3\right\}}=W_{1,\{3\}}$, this will cause $Y_{\left\{2,3\right\}}⊕Y_{\left\{1,3\right\}}=0$ from (A13). No matter what the leader central server picks, it can transmit one less codeword, denoted as $Y_{H}$, where $H\cap \hat{U}=\emptyset$, through this method.

From the transmitted codewords just mentioned, all the requesters can decode all their needed files by performing (A12). The rate $R_{\mathrm{inact}}=1/3\times 2=2/3$ and could be directly calculated by (A10).

**Remark** **A3.**
*The proposed scheme for the problem of coded caching with inactive users is with secure delivery [28] for t≥1 and r≥2, where the users who do not request and the external wiretapper cannot decode any files since the transmitted codewords only consist of the sub-files needed by requesters, and the sub-files whose index only consists of requesters would not be directly transmitted and cannot be decoded.*

## Appendix D. Order Optimality of the Adapted YWSC Scheme in Appendix Section A.1 i.e., Proof of Theorem 4

As discussed in Remark 1, in this section, under the constraint of the uncoded cache placement, we only compare the achievable rate $R_{\mathrm{adapted}-\mathrm{YWSC}}^{D_{R}}$ from (A1) with the lower bound rate for $R_{\mathrm{ave},\mathrm{req}-\mathrm{rob}}^{r*}$ and $R_{\mathrm{worst},\mathrm{req}-\mathrm{rob}}^{r*}$ from (12) and (13), respectively.

For the maximum average delivery rate, from (12), (A4), and (A10), we have that $E_{D_{R}}\left[R_{\mathrm{adapted}-\mathrm{YWSC}}^{D_{R}}\right]\geq R_{\mathrm{ave},\mathrm{req}-\mathrm{rob}}^{r*}\geq R_{\mathrm{ave},\mathrm{inact}}^{r*}=E_{D_{R}}\left[R_{\mathrm{inact}}\right]$. Furthermore, we observe that $R_{\mathrm{inact}}\geq \frac{t}{t+1}R_{\mathrm{adapted}-\mathrm{YWSC}}^{D_{R}}$ by the following:

(A15) tt+1Radapted-YWSCDR≤1t+1KK−1t−1t+1∑i=1KK−1−Ne(DK∖{i}′)tKt=Kt+1−∑i=1K1K−Ne(DK∖{i}′)K−Ne(DK∖{i}′)t+1Kt≤Kt+1−miniKK−Ne(DK∖{i}′)K−Ne(DK∖{i}′)t+1Kt≤Kt+1−K−Ne(DR)t+1Kt=Rinact,

where (A15) is because $1\leq N_{e}\left(D_{R\setminus \{i\}}\right)\leq N_{e}\left(D_{K\setminus \{i\}}^{\prime }\right)\leq N_{e}\left(D_{K}^{\prime }\right)=N_{e}\left(D_{R}\right)$ for all i∈[K].

Therefore, we have that

$$\begin{array}{cc} \; & E_{D_{R}}\left[R_{\mathrm{adapted}-\mathrm{YWSC}}^{D_{R}}\right]\geq R_{\mathrm{ave},\mathrm{req}-\mathrm{rob}}^{r*}\geq R_{\mathrm{ave},\mathrm{inact}}^{r*}=E_{D_{R}}\left[R_{\mathrm{inact}}\right]\\ \geq & \frac{t}{t+1}E_{D_{R}}\left[R_{\mathrm{adapted}-\mathrm{YWSC}}^{D_{R}}\right]\geq \frac{1}{2}E_{D_{R}}\left[R_{\mathrm{adapted}-\mathrm{YWSC}}^{D_{R}}\right]. \end{array}$$

gives the same result, which is

$$\begin{array}{cc} \; & \max_{D_{R}}R_{\mathrm{adapted}-\mathrm{YWSC}}^{D_{R}}\geq R_{\mathrm{worst},\mathrm{req}-\mathrm{rob}}^{r*}\geq R_{\mathrm{worst},\mathrm{inact}}^{r*}=\max_{D_{R}}R_{\mathrm{inact}}\\ \geq & \frac{t}{t+1}\max_{D_{R}}R_{\mathrm{adapted}-\mathrm{YWSC}}^{D_{R}}\geq \frac{1}{2}\max_{D_{R}}R_{\mathrm{adapted}-\mathrm{YWSC}}^{D_{R}}, \end{array}$$

which can be achieved for the maximum worst-case delivery rate by using similar steps. These results prove that the achievable rate $R_{\mathrm{adapted}-\mathrm{YWSC}}^{D_{R}}$ is order optimal within a factor of two under the constraint of uncoded cache placement. In addition, by the proved order optimality of the shared-link scheme within a factor of two [29] for allowing coded placement, and as discussed in Remark A2 that the only difference between the delivery scheme with inactive users and a shared-link scheme is the size of the request vector, it immediately proves that the achieved rate is within a factor of four in general and completes the proof of Theorem 4.
